# Efficacy of Berberine in Patients with Non-Alcoholic Fatty Liver Disease

**DOI:** 10.1371/journal.pone.0134172

**Published:** 2015-08-07

**Authors:** Hong-Mei Yan, Ming-Feng Xia, Yan Wang, Xin-Xia Chang, Xiu-Zhong Yao, Sheng-Xiang Rao, Meng-Su Zeng, Yin-Fang Tu, Ru Feng, Wei-Ping Jia, Jun Liu, Wei Deng, Jian-Dong Jiang, Xin Gao

**Affiliations:** 1 Department of Endocrinology and Metabolism, Zhongshan Hospital, Fudan University, Shanghai, 200032, China; 2 Institute of Materia Medica, Chinese Academy of Medical Sciences, and Peking Union Medical College, Beijing, 100050, China; 3 Department of Radiology, Zhongshan Hospital, Fudan University, Shanghai, 200032, China; 4 Department of Endocrinology and Metabolism, The Sixth People’s Hospital, Shanghai Jiaotong University, Shanghai, 200233, China; 5 Department of Endocrinology and Metabolism, The Fifth People’s Hospital, Fudan University, Shanghai, 200240, China; 6 School of public health, Fudan University, Shanghai, 200032, China; Pennington Biomedical Research Center, UNITED STATES

## Abstract

**Objectives:**

A randomized, parallel controlled, open-label clinical trial was conducted to evaluate the effect of a botanic compound berberine (BBR) on NAFLD.

**Methods:**

A randomized, parallel controlled, open-label clinical trial was conducted in three medical centers (NIH Registration number: NCT00633282). A total of 184 eligible patients with NAFLD were enrolled and randomly received (i) lifestyle intervention (LSI), (ii) LSI plus pioglitazone (PGZ) 15mg qd, and (iii) LSI plus BBR 0.5g tid, respectively, for 16 weeks. Hepatic fat content (HFC), serum glucose and lipid profiles, liver enzymes and serum and urine BBR concentrations were assessed before and after treatment. We also analyzed hepatic BBR content and expression of genes related to glucose and lipid metabolism in an animal model of NAFLD treated with BBR.

**Results:**

As compared with LSI, BBR treatment plus LSI resulted in a significant reduction of HFC (52.7% vs 36.4%, p = 0.008), paralleled with better improvement in body weight, HOMA-IR, and serum lipid profiles (all p<0.05). BBR was more effective than PGZ 15mg qd in reducing body weight and improving lipid profile. BBR-related adverse events were mild and mainly occurred in digestive system. Serum and urine BBR concentrations were 6.99ng/ml and 79.2ng/ml, respectively, in the BBR-treated subjects. Animal experiments showed that BBR located favorably in the liver and altered hepatic metabolism-related gene expression.

**Conclusion:**

BBR ameliorates NAFLD and related metabolic disorders. The therapeutic effect of BBR on NAFLD may involve a direct regulation of hepatic lipid metabolism.

**Trial Registration:**

ClinicalTrials.gov NCT00633282

## Introduction

Non-Alcoholic Fatty Liver Disease (NAFLD) is characterized by hepatic fat accumulation, insulin resistance and usually impaired glucose and lipid metabolism, which is currently a leading cause of chronic liver diseases [1]. It has been a significant health problem that affects 20–30% of the general population, among whom 5–20% developed liver cirrhosis during a 10-year period [2]. Besides, NAFLD predicts both type 2 diabetes (T2DM) and cardiovascular diseases [3], and the metabolic complex of NAFLD has attracted extensive attentions [4]. Several pharmacologic interventions have been attempted to treat NAFLD, and the agents targeting insulin resistance such as thiazolidinediones [5,6,7] have yielded promising results.

Berberine (BBR) is an active single compound isolated from Rhizoma Coptidis with a well-defined chemical structure. Recently, several studies from both clinic [8,9] and laboratory [10,11,12] reported that BBR had antidiabetic and antihyperlipidemic effects. Zhang Y et al. demonstrated that BBR had a robust glucose-lowering effect, accompanying with a significantly increase of glucose disposal rate through a randomized, double-blind, and placebo-controlled clinical trial [13]. Insulin resistance is frequently associated with hyperglycemia and dyslipidemia, and the ectopic liver fat accumulation played a key role in the development of insulin resistance [14]. In our previous study, BBR significantly decreased hepatic fat content (HFC) in high fat diet induced rats of NAFLD by reducing methylation of the MTTP promoter [15]. Therefore, we speculate that BBR may reverse many of the metabolic abnormalities associated with NAFLD by reducing the HFC. However, the effects and underlying mechanisms of BBR on hepatic steatosis and its associated metabolic abnormalities have never been investigated in patients with NAFLD.

In the present study, we carried out a randomized, multicenter, controlled, open-label clinical trial to investigate the efficacy and safety of BBR in NAFLD patients, and also explore the mechanism of BBR’s effect in an animal model of NAFLD.

## Methods

### Patients

A randomized, parallel controlled, open-label clinical trial was conducted in three medical centers for treating NAFLD patients with impaired glucose regulation (IGR) or T2DM with LSI in combination with pioglitazone (PGZ) or BBR in three centers (NIH Registration number: NCT00633282). The trial design conformed to the revised CONSORT standards for reporting randomized trials (S1 CONSORT Checklist). A schematic flow chart of the trial design is presented in S1 Fig. The planned sample size was 180 subjects, with equal assignment to each of the three study groups (60 per group). We estimated that with this sample size, the would have 90% power to detect an absolute difference in the value of HFC reduction of 7%, with a two-tailed type 1 error of 0.025.

Eligible adults were identified and recruited from unsolicited referrals to the three participating clinical centers from March 2008 to August 2011. Hepatic fat content (HFC) was assessed by a proton magnetic resonance spectroscopy (^1^H MRS) [16], and patients with a more than 13% HFC were enrolled in the study. Impaired glucose metabolism including IGR or T2DM was defined by fasting plasma glucose (FPG) value ≥ 5.6 mmol/L and/or 2 hour postload plasma glucose (PPG) following a 75-g oral glucose challenge ≥ 7.8 mmol/L. The course of T2DM should be less than 1 year for T2DM patients. Subjects were excluded if they had alcohol consumption ≥10 g/d for women and ≥20 g/d for men [17], positive for hepatitis B or C, or had other liver diseases. Patients who were treated with the following drugs within 4 weeks before enrollment were also excluded from the study, including hypoglycemic or lipid-regulating (statins, fibrates) drugs, the drugs that may impact hepatic fat content (e.g. silybin, ursodeoxycholic acid, bicyclol, phosphatidylcholine and vitamin E) and Chinese herbs. For safety concern, those with severe metabolic abnormalities and organ dysfunction were excluded, for example, HbA1c>7.5%, serum triglyceride ≥ 5.0 mmol/L, ALT or AST ≥ 2 times upper limit of normal, serum creatinine i 1.5 mg/dL (133 μmol/L) and blood pressure ≥ 160/100 mmHg in receiving lifestyle therapy and anti-hypertensive drugs. The study was approved by the ethics committee of Zhongshan Hospital, Fudan University and was conducted in accordance with the guidelines of the Declaration of Helsinki, and the Committee was in charge of monitoring the results quarterly to ensure patients’ safety and review of the therapeutic efficacy. Written informed consent was obtained from all patients.

### Study Design

Subjects who met all enrollment criteria were randomly assigned to one of the three groups for the 16-weeks clinical trial, Group A- LSI, Group B- LSI plus PGZ (15 mg q.d.) and Group C-LSI plus BBR (0.5 g, t.i.d.). BBR (berberine, Huashi Pharmaceuticals Shanghai, China, Inc.) was administered orally at a dosage of 0.5 g 30 minutes before meal, three times a day (according to the Chinese Pharmacopeia [18]). The computer-generated random allocation sequence was obtained independently by the statistician from School of public health, Fudan University, Shanghai, China. Research investigators randomized participants to one of the three arms. LSI (including dietary modification and exercise) was conducted following the standardized recommendation [19]. The daily dietary before entering the study were comparable among the three groups, and all of the participants were required to take the calorie limited-diet by subtracting 500 kcal from the mean daily calorie intake and achieve more than 150 min per week medium intensity aerobic exercise. The primary outcome was the decrease in HFC detected by ^1^H MRS; and the secondary outcomes included improvement in body weight, oral glucose tolerance test (OGTT) serum glucose and insulin, HbA1c, Homeostatic Model Assessment for Insulin Resistance (HOMA-IR), Homeostatic Model Assessment, Kunes (HOMA-β), lipid profile (TC, TG, HDL-c, LDL-c, ApoA, ApoB, ApoE, Lpa) and liver enzymes (ALT, AST, γ-GT, ALP). Both at the beginning and completion of the treatment, each participant underwent an interview by a trained investigator, an assessment of anthropometric parameters and blood examinations for the evaluation of glucose, lipid profile and liver enzymes. HFC was measured using ^1^H MRS. The patients were closely followed every 4 weeks throughout the 16-week study, and the follow-up visit was designed mainly for the assessment of safety and tolerability of the study drugs. In case that adverse event was found over the treatment course, the relevant personnel should inform responsible clinical researchers and principal investigator within 24 h. In each of the visits throughout the treatment, we evaluated interim safety related events, adherence, pill counts and collect the participants’ serum. Urine pregnancy tests were performed at each visit for female participants with child-bearing age. The serum and urine concentrations of BBR and its metabolites were measured by LC-MS/MS as described in detail previously [20].

### Measurement of liver fat content using ^1^H-MRS

LFCs were by ^1^H-MRS using a 1.5T magnetic resonance (MR) scanner (Siemens Avanto, Erlangen, Germany) equipped for proton spectroscopy acquisitions. Sagittal, coronal, and axial slices covering the whole liver were preliminarily acquired for positioning of the spectroscopy acquisition voxel. A single voxel of 8cm^3^(2 × 2 × 2 cm) was placed within the right lobe avoiding major vascular structures and subcutaneous fat tissue. The proton spectrum was acquired using the body coil after shimming over the volume of interest by means of a point-resolved spectroscopy (PRESS) sequence with the following parameters: repetition time = 1500milliseconds, echo time = 135milliseconds. Signal intensities of water peak at 4.8ppm (Sw) and the fat peak at 1.4ppm(Sf) were measured and hepatic fat percentage was calculated using the formula 100×Sf/(Sf+Sw), as described by our group previously [21].

### Animals Study

Thirty six healthy male SD rats (5–6 weeks old) weighing 190–210 gram were obtained from the Animal Development Center, Chinese Academy of Sciences, Shanghai. All rats were given free access to food and water, maintained on a 12/12-h light/dark cycle and received a high-fat diet (32.6% carbohydrate, 51.0% fat, 16.4% protein calories) for 6 weeks to establish the HFD-induced NAFLD model. BBR was purchased from Sigma-Aldrich (MO, USA), and administrated at a single dose of 200mg/kg to the HFD-induced rat of NAFLD. The rats were sacrificed by cervical dislocation, and serum and liver tissue samples were collected at 0, 4, 8, 12, 24 and 48h, respectively (n = 6 for each time point), after oral administration of single-dose BBR, which was the most commonly used dose for animal studies [11,12]. All samples were stored at -80°C. Quantitative analysis of BBR and its metabolites in blood and urine were done with the method described [22]. The frozen tissue was used for preparation of mRNA and complementary DNA for use in real-time quantitative polymerase-chain-reaction (PCR) analysis, and the details and the sequences of the primers used in this study are listed in S1 Text; Protein was also extracted from the frozen tissue, and its abundance was quantified through Western Blot Analysis. The antibodies used for immunoblotting included anti-MTTP (Bioworld), anti-Glucokinase (Proteintech), and anti- CPT1α (Proteintech). BBR and its metabolites in the liver tissue were quantified using a shimadzu triple-quadruple MS (LC–MS/MS 8040; Shimadzu Corporation, Kyoto Japan) (S1 Text). The animal study design conformed to the NC3Rs ARRIVE Guideline (S1 ARRIVE Checklist). All experimental procedures involving the use of animals were conducted in conformity with PHS policy and were approved by the Animal Use and Care Committee of Fudan University.

### Statistical Analysis

Categorical variables were normalized in frequencies (or percentages) and continuous variables were expressed as means±SD, except for skewed variables, which were presented as the median with the interquartile range given in parentheses. Kolmogorov-Smirnov test was carried out to determine the normality of the continuous variables. The difference between baseline values to that after 16 weeks on treatment with BBR plus LSI were compared with that treated with the LSI or PGZ plus LSI, using the general linear model in order to adjust for baseline value. Given the fact that there were two planned primary comparisons in the trial, P values less than 0.025 were considered to be significant. O’Brien-Fleming statistical stopping guidelines were used, with one interim analysis for efficacy performed midway through the trial. For the animals study, all the data are given as mean±s.e.m. Comparisons among different time points after BBR administration were assessed by mixed effect linear model, and Bonferroni correction was used for pairwise comparisons. A p-value<0.05 was considered statistically significant.

## Results

### Study subjects

**Fig 1:**
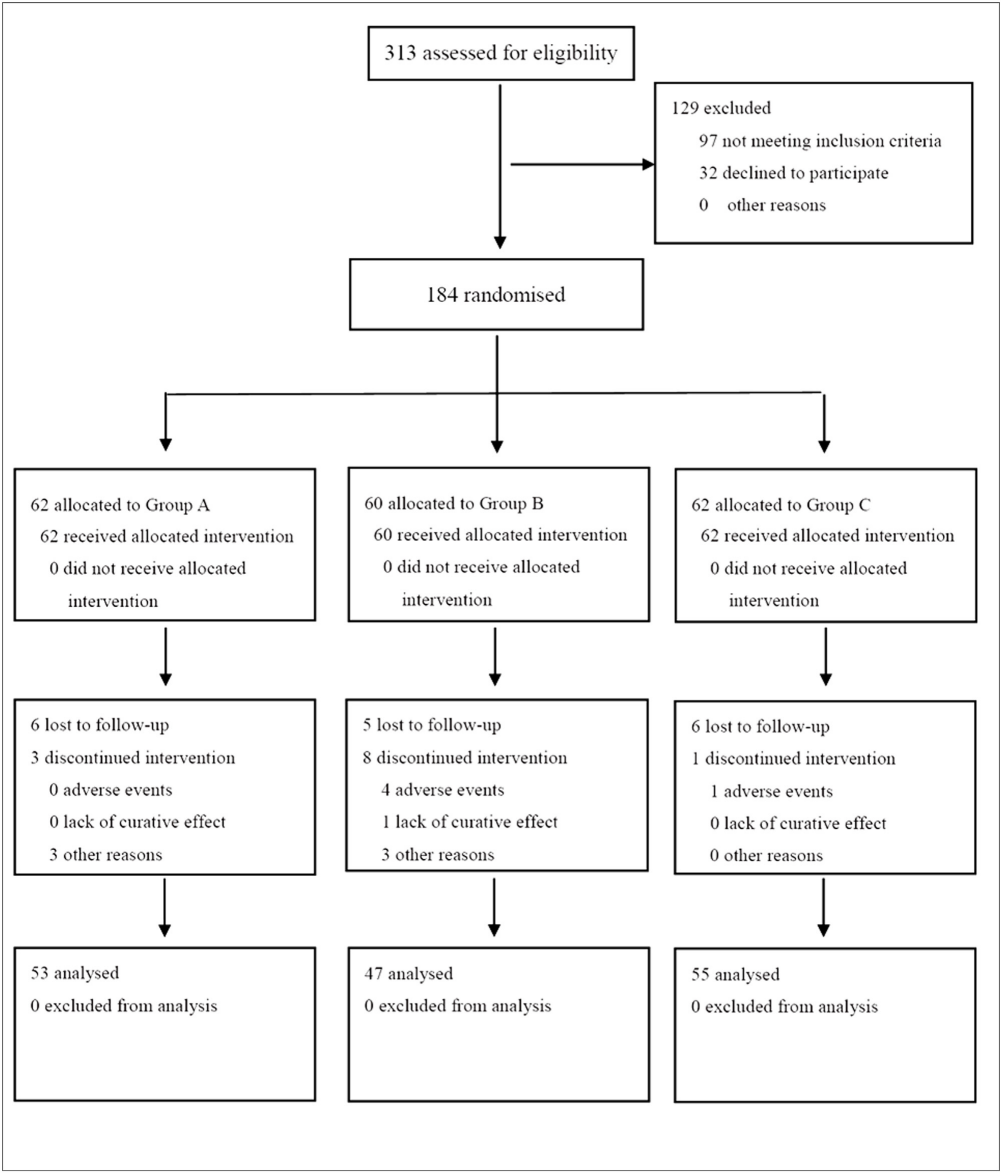
CONSORT Flow Diagram. 184 subjects were assigned to receive lifestyle intervention alone (n = 62), lifestyle intervention plus pioglitazone (n = 60), and lifestyle intervention plus berberine (n = 62). At the end of therapy, 53, 47 and 55 subjects in the three groups completed the follow-up visit, respectively.

A total of more than 313 subjects were initially enrolled for the study between March 2008 and April 2011. After clinical assessment, total 184 subjects were identified to participate the trial, of which 62 were assigned to receive LSI alone, 60 for LSI plus pioglitazone, and 62 for LSI plus BBR at randomization (Fig 1). The three groups were well matched in respect to demographic profiles, HFC and other baseline characteristics (Table 1). At the end of the trial, 53, 47 and 55 patients completed their follow-up visits, respectively (Fig 1). Compliance with the lifestyle intervention and study medications was over 90% in all groups (S1 Table).

**Table 1:** Baseline Characteristics of the Study Subjects. The data were presented as the mean±SD, except for skewed variables, which were presented as the median with the interquartile range given in parentheses.

	LSI	LSI plus PGZ	LSI plus BBR
Sex (M/F)	32/30	28/32	38/24
Age (year)	50.64±10.69	53.52±8.62	50.72±9.76
Weight (kg)	75.73±11.13	74.98±12.73	78.71±15.99
BMI(kg/m2)	27.27±2.80	27.47±3.74	28.08±4.17
Waist (cm)	93.34±7.81	93.09±8.91	95.88±10.98
HFC (%)	29.5(21.0–44.5)	29.8(20.5–44.0)	30.2(22.3–43.1)
Serum glucose (mmol/L)			
0min	6.09±0.96	6.28±1.08	6.37±0.92
30min	10.61±1.91	11.14±1.88	11.10±1.54
60min	12.36±2.93	12.87±3.01	12.98±2.59
120min	9.97±3.17	11.18±3.54	11.11±2.98
180min	6.23±2.42	6.78±2.85	6.92±2.50
AUCg	39.11±8.42	41.73±9.07	41.84±7.52
HbA1c(%)	6.17±0.67	6.42±0.68	6.46±0.70
Serum insulin (mU/mL)			
0min	15.0(9.3–18.6)	13.7(10.0–18.2)	13.6(8.9–17.4)
30min	66.0(38.2–89.4)	58.6(33.5–78.4)	52.0(36.2–68.4)
120min	83.5(59.2–132.1)	88.8(58.9–136.6)	81.4(49.1–113.1)
HOMA-IR	4.22±2.51	4.26±2.47	4.20±2.85
HOMA±	131.08±87.58	123.85±62.95	119.41±114.32
ΔI30/ΔG30	13.51±11.65	10.94±8.99	10.06±8.79
Lipid profile			
TC (mmol/L)	4.94±0.71	5.38±0.89	5.29±0.91
TG (mmol/L)	1.93±0.70	2.16±0.91	2.19±1.10
HDL-c (mmol/L)	1.20±0.25	1.19±0.25	1.16±0.26
LDL-c (mmol/L)	2.91±0.68	3.25±0.94	3.23±0.85
APO-A (g/L)	1.27(1.11–1.39)	1.30(1.18–1.54)	1.25(1.08–1.44)
APO-B (g/L)	1.00±0.19	1.08±0.20	1.07±0.21
APO-E (mg/L)	46(39–52)	46(39–58)	49(40–57)
LPa (mg/L)	135(90–219)	142(79–233)	105(54–183)
Liver enzyme (U/L)			
ALT	34(20–54)	41(26–65)	33(23–49)
AST	25(20–30)	28(20–43)	24(19–32)
γ-GT	36(22–60)	40(27–58)	40(27–69)

### Hepatic fat content and liver enzymes

**Fig 2:**
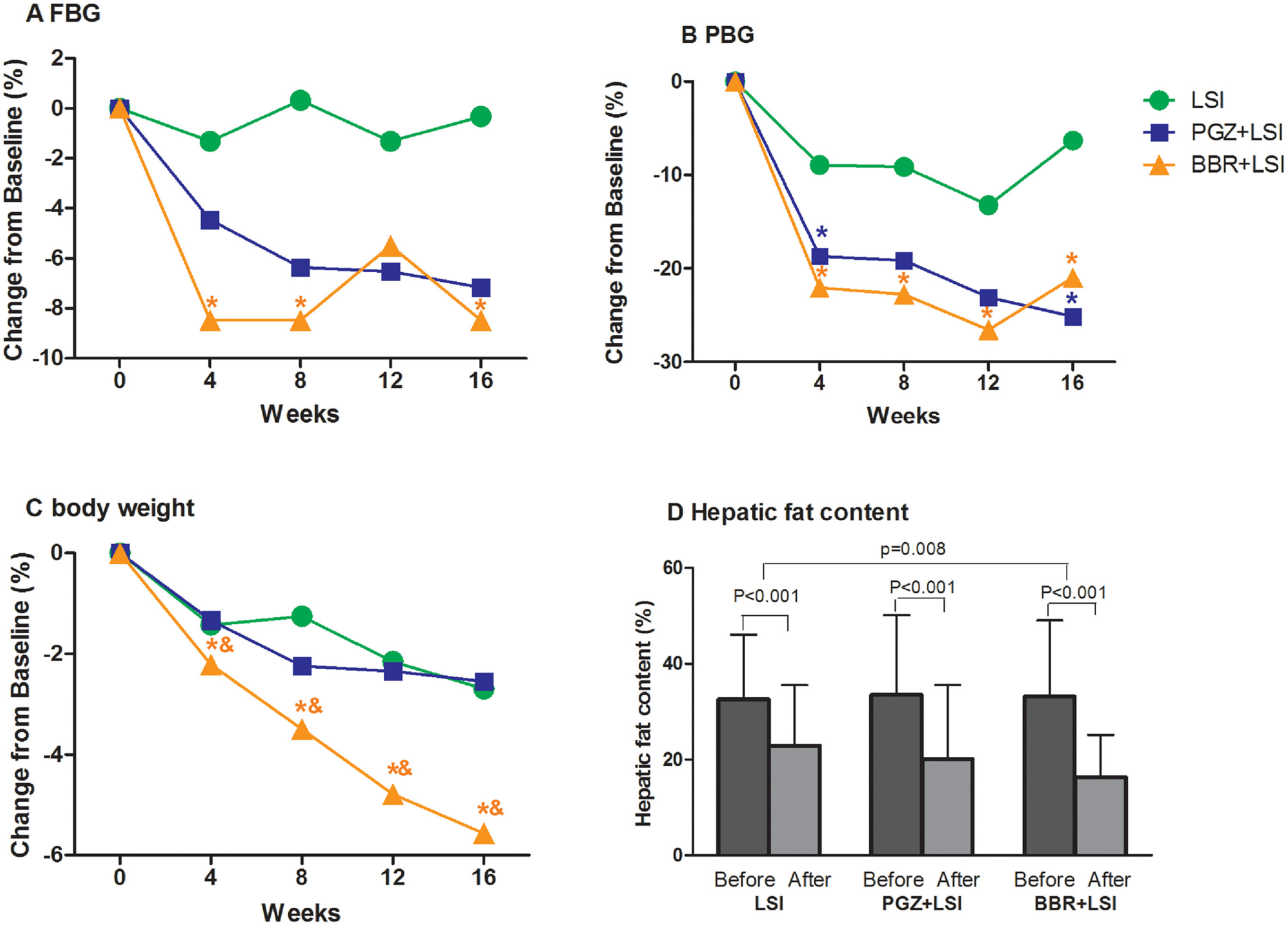
Reduction of blood glucose, body weight and hepatic fat content after therapy. Mean values are shown for percentage changes from baseline of A) FBG, B) PBG, C) body weight and D) The mean HFC of the three groups before and after treatment. *p<0.05, compared with LSI group, &p<0.05, comared with LSI plus PGZ group.

After treatment, HFC decreased by 57.2% in the LSI plus BBR group (P<0.001) and by 36.4% in the LSI group (P<0.001). Treatment with LSI plus BBR caused more reduction of HFC as compared to that with LSI alone (p = 0.008, Fig 2). Moreover, the effect of BBR on HFC was no smaller than that of PGZ (15mg per day) (p = 0.054) [23]. Liver enzymes were also reduced in all groups after treatment, and the reduction of ALT and AST showed no significant difference between the LSI plus BBR and LSI plus PGZ groups at the 16^th^ week (p = 0.855 and p = 0.632, respectively) (Table 2).

**Table 2:** Changes of clinical and biochemical parameters after treatment. All parameters were adjusted for age, BMI and the baseline parameter and represented as means (95%CI). P value after adjustment for age, BMI, baseline data.

	LSI	LSI plus PGZ	LSI plus BBR	P value (LSI plus BBR vs. LSI)	P value (LSI plus BBR vs. LSI plus PGZ)
Number	53	47	55	-	-
Weight (kg)	-1.99(-2.76~-1.23)	-1.94(-2.75~-1.12)	-4.29(-5.04~-3.54)	<0.001	<0.001
BMI(kg/m^2^)	-0.72(-0.99~-0.44)	-0.67(-0.97~-0.38)	-1.51(-1.79~-1.25)	<0.001	<0.001
Waist (cm)	-2.14(-3.03~-1.26)	-2.60(-3.55~-1.66)	-4.84(-5.70~-3.97)	<0.001	0.001
HFC(%)	-11.4(-14.8~-7.9)	-12.1(-15.8~-8.5)	-17.4(-20.8~-14.0)	0.008	0.054
Blood glucose (mmol/L)					
0min	-0.15(-0.41~-0.12)	-0.38(-0.66~-0.10)	-0.47(-0.73~-0.20)	0.096	0.658
30min	-0.63(-1.06~-0.19)	-0.91(-1.38~-0.45)	-1.03(-1.46~-0.60)	0.196	0.725
60min	-1.23(-1.80~-0.65)	-1.18(-1.79~-0.57)	-1.86(-2.42~-1.29)	0.125	0.112
120min	-1.05(-1.73~-0.37)	-2.44(-3.17~-1.72)	-2.19(-2.87~-1.51)	0.020	0.609
180min	-0.90(-1.35~-0.44)	-1.48(-1.97~-1.00)	-1.57(-2.02~-1.11)	0.042	0.799
AUCg	-3.33(-4.93~-1.73)	-5.51(-7.21~-3.81)	-6.14(-7.72~-4.55)	0.015	0.594
HbA1c(%)	-0.30(-0.43~-0.16)	-0.44(-0.58~-0.30)	-0.47(-0.60~-0.33)	0.078	0.793
Serum insulin (mU/mL)					
0min	-0.85(-2.43~0.72)	-1.66(-3.35~0.02)	-1.59(-3.14~-0.03)	0.515	0.949
30min	-3.10(-11.54~5.35)	1.24(-7.73~10.22)	6.93(-1.35~15.20)	0.098	0.362
120min	4.5(-10.9~19.9)	-15.4(-32.0~1.1)	-13.6(-28.9~1.7)	0.102	0.874
HOMA-IR	-0.26(-0.72~0.20)	-0.69(-1.18~-0.20)	-1.06(-1.51~-0.61)	0.014	0.298
Lipid profile					
TC(mmol/L)	-0.12(-0.31~0.07)	-0.11(-0.31~0.10)	-0.52(-0.71~-0.33)	0.004	0.004
TG(mmol/L)	-0.02(-0.24~0.21)	-0.12(-0.36~0.12)	-0.45(-0.68~-0.23)	0.007	0.050
HDL-c(mmol/L)	0.005(-0.034~0.044)	0.065(0.023~0.107)	0.010(-0.029~0.049)	0.861	0.061
LDL-c(mmol/L)	-0.14(-0.32~0.04)	-0.05(-0.25~0.14)	-0.24(-0.42~-0.07)	0.442	0.154
APO-A(g/L)	-0.01(-0.07~0.05)	-0.00(-0.06~0.07)	-0.09(-0.15~-0.03)	0.055	0.036
APO-B(g/L)	-0.05(-0.09~-0.01)	-0.05(-0.09~-0.00)	-0.12(-0.16~-0.08)	0.022	0.023
APO-E(mg/L)	-7.0(-10.7~-3.2)	-8.9(-13.0~-4.8)	-10.9(-14.6~-7.2)	0.143	0.476
LP(a)(mg/L)	4.53(-22.3~31.3)	17.8(-11.2~46.9)	23.6(-2.9~50.1)	0.319	0.774
Liver enzyme (U/L)					
ALT	-14.1(-18.0~-10.2)	-20.5(-24.8~-16.2)	-21.0(-24.9~-17.1)	0.015	0.855
AST	-6.5(-8.2~-4.8)	-8.3(-10.2~-6.4)	-8.9(-10.7~-7.2)	0.050	0.632
γ-GT	-8.4(-13.9~-3.0)	-14.3(-20.2~-8.5)	-11.7(-17.1~-6.3)	0.403	0.517

### Responses in energy metabolism

In comparison with LSI alone, BBR exhibited extra decreases in body weight [-4.29(-5.04 –-3.54)kg vs -1.99(-2.76 –-1.23)kg, p<0.001], BMI [-1.51(-1.79 –-1.25)kg/m^2^ vs -0.72(-0.99 –-0.44) kg/m^2^, p<0.001], waist circumference[-4.84(-5.70 –-3.97)cm vs -2.14(-3.03 –-1.26)cm, p<0.001], HFC [-17.4(-20.8 –-14.0)% vs -11.4(-14.8 –-7.9)%, p = 0.008), 2h postload glucose [-2.19(-2.87 –-1.51)mmol/L vs -1.05(-1.73 –-0.37)mmol/L, p = 0.020), area under the OGTT curve [-6.14(-7.72 –-4.55) vs -3.33(-4.93 –-1.73), p = 0.015], HOMA-IR [-1.06(-1.51 –-0.61) vs -0.26(-0.72–0.20), p = 0.014], serum cholesterol [-0.52(-0.71 –-0.33)mmol/L vs -0.12(-0.31–0.07)mmol/L, p = 0.004], APO-B [-0.12(-0.16 –-0.08)g/L vs -0.05(-0.09–0.01)g/L, p = 0.022] and triglycerides [-0.45(-0.68 –-0.23)mmol/L vs -0.02(-0.24–0.21)mmol/L, p = 0.007](Table 2), showing clearly a significant benefit of BBR in metabolism. The blood glucose reduction in LSI plus BBR group occurred in the first 4 weeks of treatment and was sustained throughout the trial; a remarkable and time-dependent decrease of body weight was also observed in this group (Fig 2). As compared with the PGZ plus LSI group (the positive reference of the study), BBR plus LSI showed comparable effects in control of blood glucose and insulin sensitivity, whereas BBR has additional benefits in body weight and serum lipids (Table 2).

### Adverse Events

A total of 61 drug-related adverse events (AEs) occurred in the participants. The most common AEs related to BBR were anorexia and upset stomach (30.95% of BBR-related AEs), diarrhea (26.19%) and constipation (14.29%), which could be well-tolerated within the initial two weeks of the study. No serious AEs, such as congestive heart failure, bone fractures, liver toxicity, were observed in the subjects. AEs occurred in PGZ group mainly included muscle pain, fatigue, and cardiac symptoms (S2 Table). Owing to the AEs four patients discontinued the treatment with PGZ and one with BBR.

### Serum and urine BBR concentrations

Eleven subjects from the LSI plus BBR group and eleven subjects from the LSI group were randomly selected for measurement of concentrations of serum and urine BBR and its metabolites before and after the 16-week intervention. At the end of 16-week treatment, the median levels of BBR in serum and urine were 6.99ng/ml and 79.2ng/ml, respectively, in the BBR-treated subjects (Table 3), whose baseline BBR concentrations were not detectable. In contrast, BBR was also not detectable before and after the intervention in LSI group. Urine analysis showed that BBR was mainly excreted as its prototype with concentrations ranging from 4.49 ng/mL to 645.48 ng/mL. Although five BBR metabolites (M1, M2, M4, M12, M13) were detected in urine of the patients, all of the metabolites showed very low concentrations raging from 0.01 to 10.15 ng/mL (approximately 70-fold lower than BBR). All the results indicated that the botanic compound BBR was well absorbed, metabolized and excreted mainly as its prototype from the urine.

**Table 3:** Contents of BBR and its metabolites in human blood and urine.

	Compound	M.W (*m/z*)	Concentration (ng/mL) [Median (P_25_-P_75_)]
Serum	BBR	336	6.99(4.65–9.82)
Urine	BBR	336	79.16 (3.37–326.16)
	M1	322	0.56 (0–3.01)
	M2	322	0.66 (0–2.04)
	M4	338	0.03(0–0.08)
	M12	498	0(0–0.02)
	M13	402	0.17(0–0.57)

### BBR and Its Metabolites in liver after Oral Administration in Animal Model

**Fig 3:**
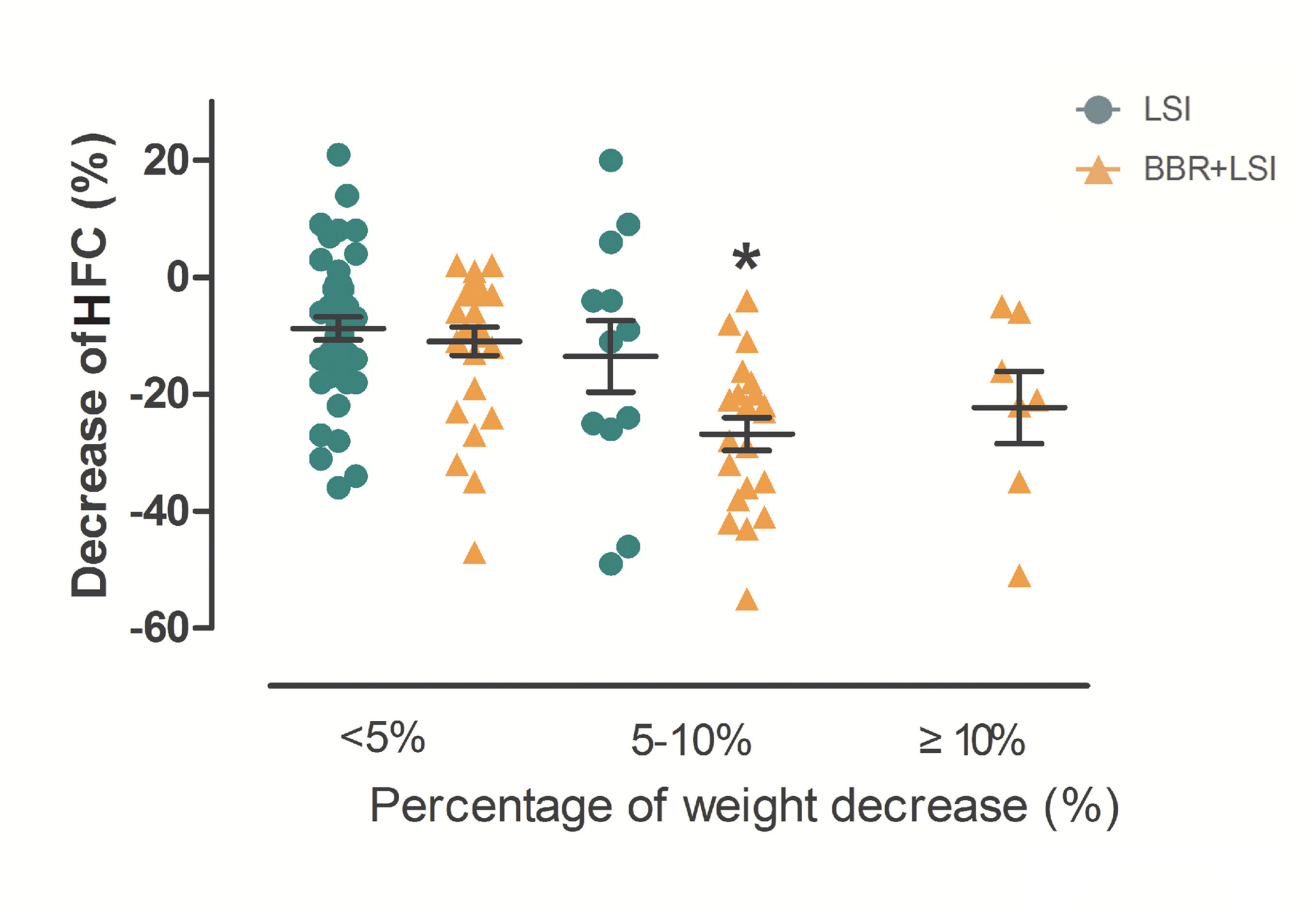
Comparison of HFC decrease (%) between the LSI and LSI plus BBR groups at different degrees of body weight change (<5%, 5–10% and ≥10% body weight change). *p<0.05, compared with the LSI group with the same degree of weight loss.

**Fig 4:**
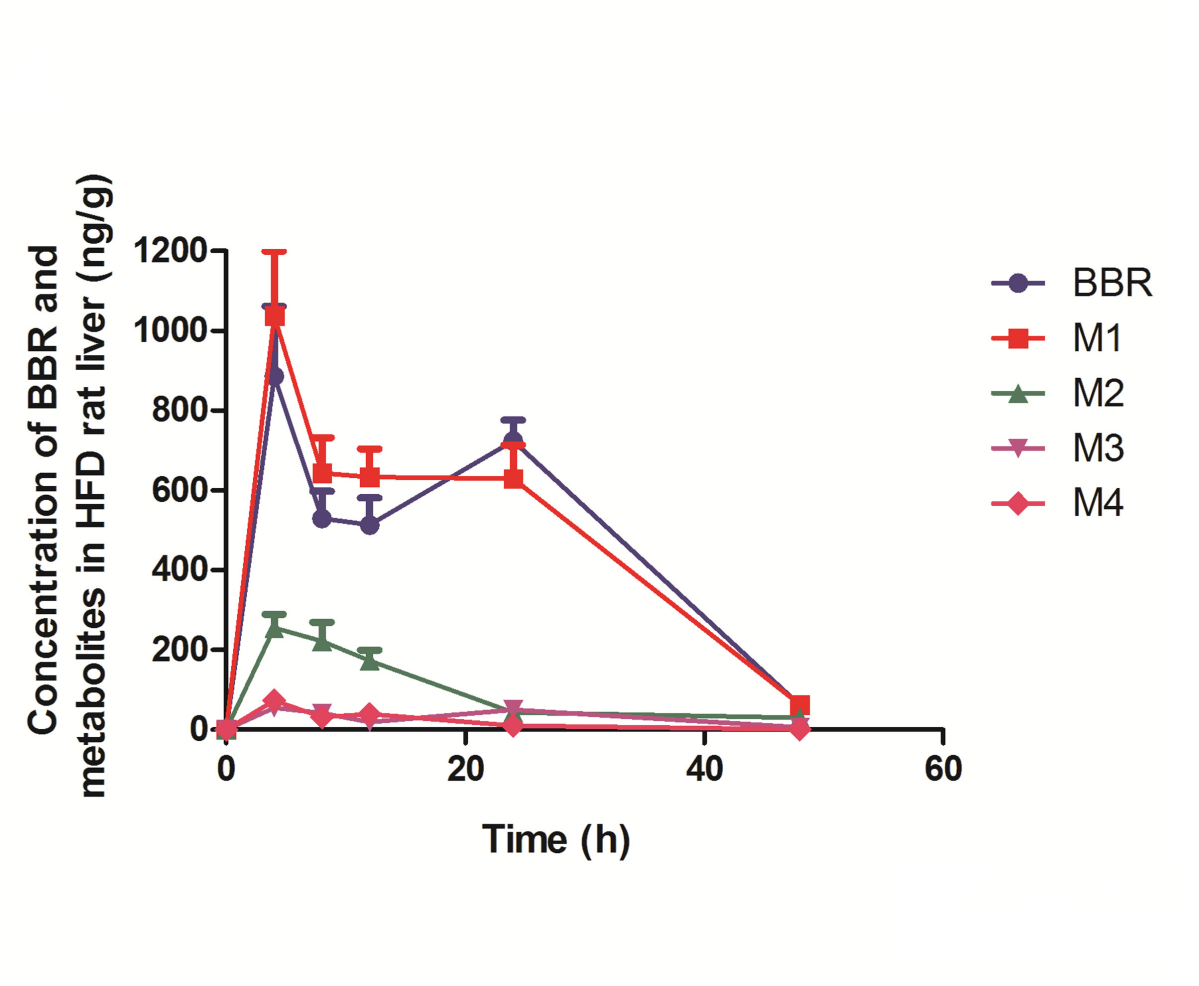
Concentration of BBR and its metabolites (M1, M2, M3, M4) in HFD rat liver and plasma (Mean ± SEM, n = 6).

It is noticeable that Subjects at the BBR plus LSI group lost significantly more hepatic fat content than the LSI group with the same degree of body weight loss (Fig 3), which indicated that the benefits of BBR on NAFLD and its related metabolic diseases might involve a direct action on hepatic energy metabolism. To further understand the therapeutic effects of BBR, we conducted experiments in a HFD-induced NAFLD animal model by treating rats with a single dose of BBR to exclude the interference of body weight change. As shown in Fig 4, BBR and its metabolites were distributed in the liver. Moreover, BBR concentrations in rat liver were 50 times higher than that in the plasma (Table 4). The first peak of BBR (886.80 ±174.55ng/g) in the liver occurred at 4 hrs after oral administration of the drug and second peak at 24 hrs (724.44±51.89 ng/g), followed by a significant decline. BBR metabolites exhibited a similar time-concentration relationship to that of BBR.

**Table 4:** Concentration of BBR in HFD rat liver and plasma.

Time point	Liver Concentration (ng/ml)	Plasma Concentration (ng/ml)
0h	0	0
4h	782.37(362.51–1132.86)	0.78(0.00–2.74)
8h	472.11(295.77–660.08)	0.30(0.00–1.39)
12h	576.76(380.19–769.51)	6.77(0.40–31.40)
24h	734.46(486.33–921.22)	3.24(1.05–26.56)
48h	51.71(24.35–109.61)	4.14(3.75–5.32)

### Effects of BBR on Hepatic Gene Expression

**Fig 5:**
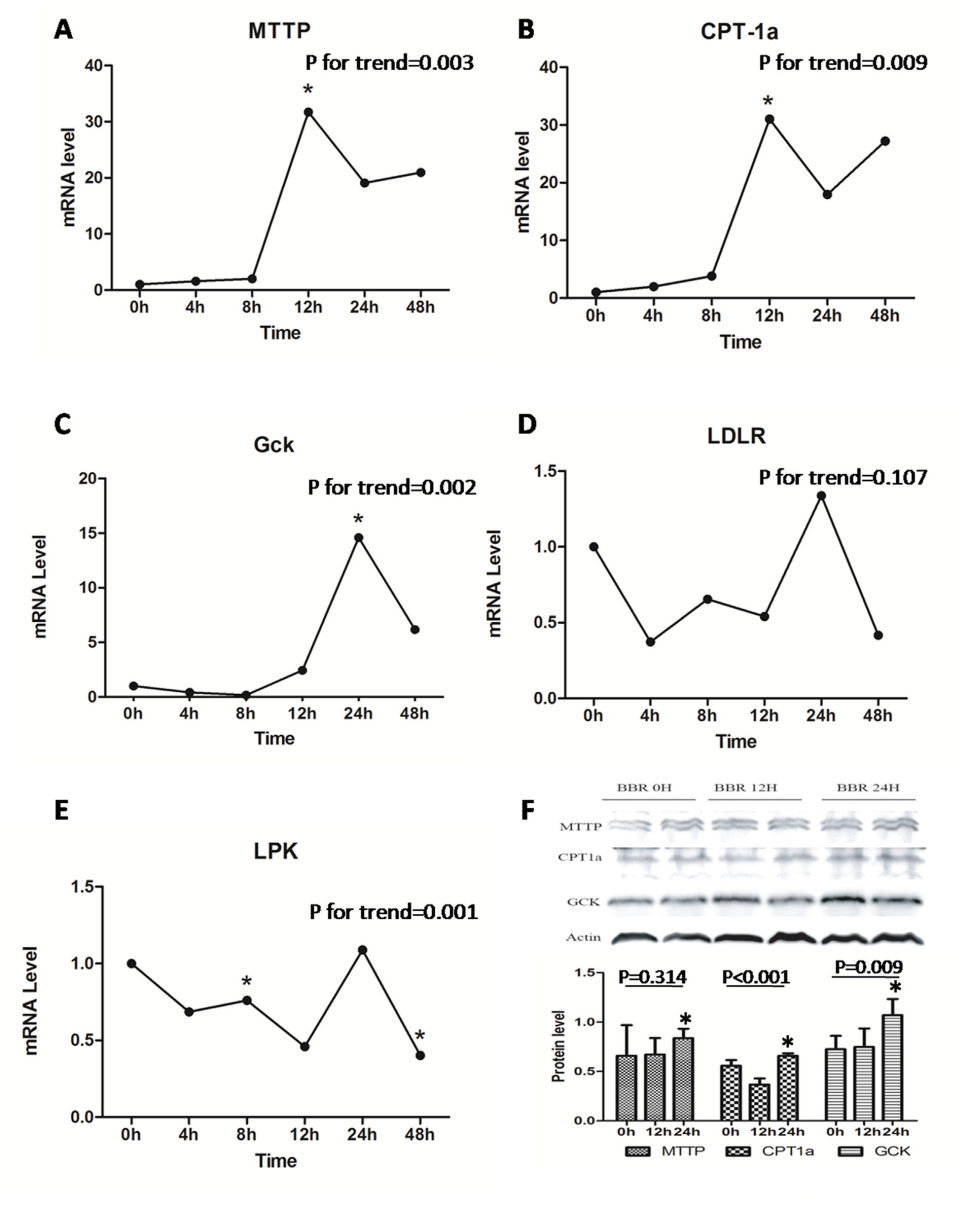
A-E) Altered expression of genes closely related to glucose and lipid metabolism in liver of SD rats. The samples were examined within 48h after single-dosing of BBR in oral route. Real-time quantitative PCR was used to detect the liver A) MTTP, B) CPT-1α, C) GCK, D) LDLR and E) LPK mRNA expression at different time courses. F) Quantification of the MTTP, CPT-1, C) GCK, D) LDLR and E) LPK mRNAes were examined within 48h after single-dosing of BBR in oral route. liMTTP, microsomal triglyceride transfer protein; CPT-1α, carnitine palmitoyltransferase-1α; GCK, glucokinase; LDLR, low density lipoprotein receptor; LPK, liver pyruvate kinase.

Having shown a preferable distribution of BBR in the liver, we further determine the effect of BBR on hepatic glucose and lipid metabolism. One single dose of BBR treatment significantly increased serum triglyceride and reduced serum ALT and AST concentration within 48hours, without change of body weight (Table 5). As shown in Fig 5A–5E, the relative mRNA of CPT-1α, MTTP and GCK were significantly up-regulated (P<0.05) in the liver of HFD-fed rat orally treated with a single dose of BBR. Western blot analysis showed a result consistent with that in real-time PCR (Fig 5F). The expression of CPT-1, MTTP and GCK reached the peak at 24 hrs after oral administration of BBR, parallel with a second peak of liver BBR concentration (Table 4). These data suggest a pleiotropic effect of BBR on the energy metabolism network in the liver, which may account for its direct therapeutic effect on NAFLD.

**Table 5:** Phenotype of HFD-induced NAFLD rats after treating with single-dose BBR.

	HFD-0h	HFD-4h	HFD-8h	HFD-12h	HFD-24h	HFD-48h	P for trend
**Body weight (g)**	428.50±20.79	391.00±20.15	400.00±39.59	426.67±21.96	429.00±35.38	402.50±31.50	0.103
**Liver weight (g)**	13.96±0.90	12.42±1.26	13.13±1.50	13.96±1.28	14.46±2.46	12.09±1.79	0.096
**Brown fat (g)**	0.47±0.14	0.31±0.07	0.39±0.16	0.41±0.10	0.42±0.08	0.37±0.06	0.227
**Epididymal fat (g)**	6.38±3.11	4.46±0.67	5.34±1.73	5.72±1.75	6.09±1.78	5.40±1.51	0.587
**TC**	1.64±0.34	1.73±0.24	1.89±0.46	1.69±0.51	2.04±0.36	2.12±0.32	0.177
**TG**	0.36±0.08	0.70±0.23	0.49±0.19	0.34±0.09	0.70±0.28	0.83±0.17	<0.001
**HDL-c**	0.90±0.19	0.93±0.19	1.09±0.28	0.97±0.31	1.09±0.21	1.08±0.19	0.551
**ALT**	92.67±52.79	47.17±5.91	39.33±7.23	46.17±6.59	79.17±18.71	75.67±17.0	0.002
**AST**	376.67±197.57	143.83±13.18	126.17±24.26	139.17±24.98	270.33±41.00	297.83±89.60	<0.001

## Discussion

The beneficial effects of BBR on glucose and lipid metabolism have been fully demonstrated. However, the mechanism underlying its therapeutic effect is still unclear. In our current study, we found that BBR was absorbable and mainly located in the liver (50 times higher than that in the plasma) after oral administration. With its preferential distribution in liver, BBR profoundly ameliorated liver steatosis in the NAFLD patients from our randomized clinical trial and directly regulated the expression of hepatic genes related to glucose and lipid-metabolism. To the best of our knowledge, our current study is the first human study focusing on the BBR’s therapeutic effects on NAFLD, and we also measure its concentration in serum, urine and liver as well as hepatic gene expression related to glucose and lipid metabolism after BBR treatment.

In the present study, BBR treatment for 16-weeks in combination with LSI significantly reduced hepatic fat content in NAFLD patients, paralleled with a global metabolic benefit as reflected in reducing body weight, and improving glucose and lipid profiles. In comparison with LSI alone, BBR exhibited extra benefits in the improvement of body weight, HFC, HOMA-IR and lipid levels. Even compared with PGZ (15 mg/day) [24], BBR had not only a similar reduction of blood glucose and HFC, but also beneficial effects on body weight. Although gastro-enteric AEs related to BBR were observed, they were mild and tolerable. BBR was absorbable after oral administration in our study patients and studies on rat models showed that BBR was located favorably in the liver and could alter hepatic metabolism-related gene expression.

Patients in the berberine group lost significantly more liver fat content, and showed more reductions in blood glucose, triglycerides and cholesterol than the LSI group, which was concordant with those in previous studies [8,9,13]. A remarkable decrease in body weight was also obeserved in participants of BBR group. Several studies reported that the BBR had an extreme low bioavailability of less than 1% in BBR-treated animals [25,26], therefore it was believed that BBR was not absorbable in human gastrointestinal tract and its beneficial effects on hepatic fat, insulin resistance as well as glucose and lipid metabolism mainly depended on its effect on gut microbiota[27], and all of the hepatic fat and metabolic improvements might depended on significant weight loss after BBR treatment. However, our current study found that subjects at the BBR plus LSI group lost significantly more liver fat content than the LSI group with the same degree of body weight reduction (Fig 3), which indicated that the improvement of liver steatosis after BBR treatment not only related to the significant body weight reduction. Therefore, we further analyzed the serum and urine BBR concentrations using the accurate LC-MS/MS analysis in our human study, and found that BBR was absorbed by oral administration, metabolized in the liver and excreted in urine mainly in its prototype using LC-MS/MS analysis, which suggested its direct effect on the liver.

To further explore the possible mechanism underlying BBR’s direct effect on NAFLD in human beings, we measured the distribution of BBR (and its metabolites) after BBR treatment in the HFD-induced animal model of NAFLD. As compared to its concentration in blood, BBR (and its metabolites) favored to locate in liver with a concentration 50 times higher than that in the plasma (Table 4). In fact the phenomena of liver-selective enrichment have been reported in BBR[20,28] and other botanic medicinal alkaloids[29].

However, the liver-selective enrichment of BBR is possibly the hepatic first-pass effect, which does not mean a bioactivity on glucose and lipid metabolism, so we examined hepatic expression of a group of energy metabolism related genes MTTP, CPT-1a and GCK at 0, 4, 8, 12, 24 and 48h after oral administration of a single dose of BBR. The expression of these genes in liver was significantly up-regulated after BBR treatment, with no significant change of body weight at each time point, indicating a direct effect of BBR on hepatic expression of metabolism related genes. MTTP was for the assembly and secretion of apoB-containing lipoproteins (VLDL and LDL)[15], CPT-1a was a part of the outer membrane fatty acid transfer complex and catalyzed the primary regulated step in overall mitochondrial fatty acid oxidation[30]. The up-regulation of these genes after BBR treatment might promote the export and β-oxidation of liver fat, and partially account for its therapeutic effect in its therapeutic effect in improving liver steatosis. GCK was for regulation of glucose metabolism rate[31], and also increased after BBR treatment. It has been reported that BBR could decrease insulin resistance by activating liver AMPK [32], and hepatic GCK up-regulation might relate to the activation of hepatic AMPK pathway[33]. Therefore, BBR may have multiple effects on liver genes associated with lipid or glucose metabolism. It is likely that the significant anti-NAFLD effect of BBR is related to its favorite location in liver and its direct effects on multiple hepatic genes that links to energy metabolism.

The limitation of this study is that none of these patients was examined by liver biopsy because of the ethics concern, and the effects of BBR on human hepatic histological inflammation, fibrosis as well as the genes related to energy metabolism need to be further studied.

## Conclusion

Oral administration of BBR significantly reduced HFC, body weight, and improved metabolic profile for lipid and glucose in patients with NAFLD. The therapeutic efficacy of BBR on NAFLD and its related glucose and lipid metabolism related to its favorite location in liver and its direct effects on multiple hepatic genes that links to energy metabolism. Therefore, BBR is a promising agent to treat NAFLD, as well as their related metabolic diseases.

## Supporting Information

S1 ARRIVE Checklist NC3Rs ARRIVE Guidelines. (PDF)

S1 CONSORT Checklist CONSORT Checklist. (DOC)

S1 Fig Design schematic of the clinical trial. (TIF)

S1 Protocol Trial Protocol (English). (DOCX)

S2 Protocol Trial Protocol (Chinese). (DOC)

S1 Table Compliance of lifestyle intervention and medication in the three groups. (DOCX)

S2 Table Drug-related adverse events in the clinical trial. (DOCX)

S1 Text Detailed description of the methods of real-time quantitative polymerase-chain-reaction (PCR) analysis, Western blot analysis, and measurement of BBR and its metabolites in the liver tissue using a shimadzu triple-quadruple MS (LC–MS/MS 8040; Shimadzu Corporation, Kyoto Japan) in animal model. (DOCX)
