# Supplementary figures and images for: Efficacy of Berberine in Patients with Non-Alcoholic Fatty Liver Disease
Source: PLoS One. 2015 Aug 7;10(8):e0134172. doi: 10.1371/journal.pone.0134172 (PMC4529214; doi:10.1371/journal.pone.0134172)

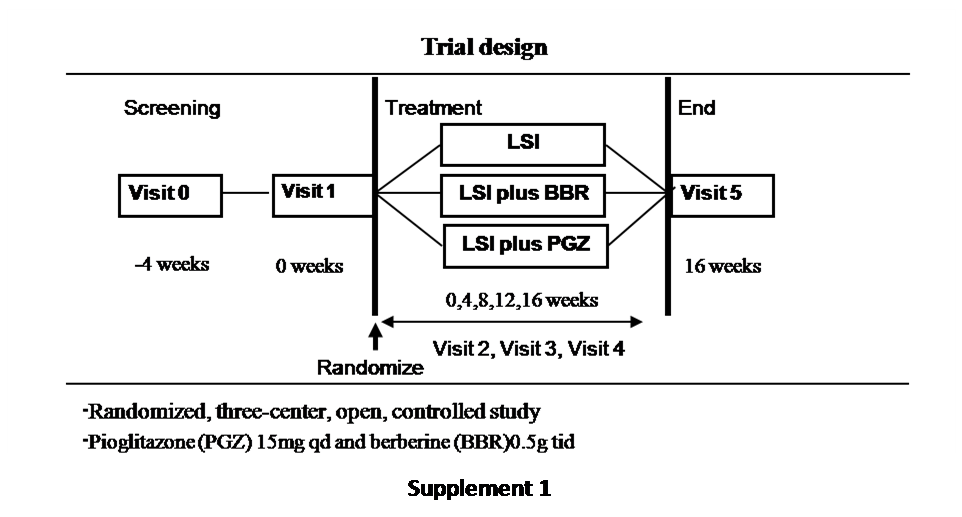

Supplement: S1 Fig — (TIF) [file pone.0134172.s003.tif]
