# Supplementary material for: Efficacy of Berberine in Patients with Non-Alcoholic Fatty Liver Disease
Source: PLoS One. 2015 Aug 7;10(8):e0134172. doi: 10.1371/journal.pone.0134172 (PMC4529214; doi:10.1371/journal.pone.0134172)
Supplement: S1 Protocol — (DOCX) [file pone.0134172.s004.docx]

**Role of Pioglitazone and Berberine in the Treatment of Non-alcoholic Fatty Liver Disease(NAFLD) Patients With Impaired Glucose Regulation or Type 2 Diabetes Mellitus**

**Final Study Protocol**

**Supported by**

**Shanghai Municipal Science and Technology Commission (07JC14011)**

**Principal Investigator**

**Xin Gao, MD, Professor of Endocrinology and Metabolism**

**Zhongshan Hospital, Fudan University, Shanghai, China**

**Aug. 8^th^, 2008**

**Table of Contents**

**PROTOCOL SYNOPSIS .........................................................................................44**

**1 STUDY FLOWCHARTS ..................................................................................... 50**

1.1 **Trial Design** ..........................................................................................................50

**1.2 Schedule of Activities and Assessments** .............................................................51

**2 BACKGROUND AND RATIONAL ................................................................... 52**

**2.1 The burden of non-alcoholic fatty liver disease and associated metabolic disorders in China**.......................................................................................................52

**2.2 Pathogenesis and potential treatments of NAFLD .**......................................... 53

**3 STUDY OBJECTIVES..........................................................................................54**

**3.1 Primary**................................................................................................................ 54

**3.2 Secondary**............................................................................................................. 55

**4 STUDY DESIGN AND MANAGEMENT OVERVIEW ..................................55**

**4.1 Study Design Overview**....................................................................................... 55

**4.2 Study Organization** ............................................................................................ 56

**4.3 Outcomes**.............................................................................................................. 59

**4.4 Specimen banking**................................................................................................ 59

**5 PARTICIPANT SELECTION ............................................................................. 59**

**5.1 Inclusion criteria** ................................................................................................ 60

**5.2 exclusion criteria** ................................................................................................ 60

**6 TREATMENTS ..................................................................................................... 62**

**7 STUDY DRUG HANDLING ................................................................................ 63**

**7.1 Supply and storage** ............................................................................................. 63

**7.2 Dispense and management** ................................................................................ 63

**7.3 Prohibited Concomitant Treatment**.................................................................. 64

**7.4 Treatment Accountability and Compliance....**.................................................. 64

**7.5 Treatment Discontinuation**................................................................................. 64

7.5.1 Temporary treatment discontinuation with investigational product................... 64

7.5.2 Definition treatment discontinuation with investigational product(s)............... 65

7.5.3 Handling of patients after definitive treatment discontinuation........................ 65

**8 SAFETY ……………………................................................................................. 66**

**8.1 Adverse event monitoring** .................................................................................. 66

**8.2 Definition of adverse event and serious adverse event** .................................... 66

**8.3 Adverse event recording** .................................................................................... 68

**8.4 Adverse event treatment** .................................................................................... 69

**8.5 Follow-up** ............................................................................................................. 70

**9 STUDY PROCEDURES ....................................................................................... 70**

**9.1 Screening (V0)** ..................................................................................................... 71

**9.2 Visit 1 (V1) .**.......................................................................................................... 71

**9.3 Visit 2 (V2) .**.......................................................................................................... 72

**9.4 Visit 3 (V3) .**.......................................................................................................... 73

**9.5 Visit 4 (V4) .**.......................................................................................................... 74

**9.6 Visit 5 (V5) .**.......................................................................................................... 75

**10 STATISTICAL CONSIDERATIONS ............................................................... 76**

**10.1 Statistical Analysis Plans.**.................................................................................. 76

**10.2 Sample Size Estimation.**.................................................................................... 76

**10.3 Statistical Analyses.**........................................................................................... 77

**11 ETHICAL AND REGULATORY STANDARDS…....................................... 78**

**11.1 Ethical Principles**............................................................................................... 78

**11.2 Laws and regulations**......................................................................................... 78

**11.3 Informed Consent**.............................................................................................. 78

**12 REFERENCES..................................................................................................... 79**

**Protocol Synopsis**

| **Title:** | Role of Pioglitazone and Berberine in the Treatment of Non-alcoholic Fatty Liver Disease(NAFLD) Patients With Impaired Glucose Regulation or Type 2 Diabetes Mellitus |
| --- | --- |
| **Primary Objective:** | To assess the effects and safety of pioglitazone (15mg daily), a thiazolidinedione insulin sensitizer, and Berberine (BBR), a component isolated from a Chinese herb, on hepatic fat content and glucose and lipid metabolism in NAFLD patients with impaired glucose regulation (IGR) or T2DM. |
| **Study Design:** | The study is a three-center, randomized, parallel controlled, open clinical trial of treatment with pioglitazone, berberine or no drug on the basis of life style intervention for NAFLD patients with IGR or T2DM. The screening period for evaluating eligibility and collecting baseline data lasted up to 4 weeks before randomization. Eligible patients were randomized to receive pioglitazone (15 mg q.d), berberine (0.5g t.i.d.) or no-drug on basis of Life style intervention for 16 weeks. The primary comparison will be made using an intention-to-treat analysis of the change in OGTT (glucose, insulin, AUC of glucose), HbA1c; lipid profile（TC, TG, HDL-c, LDL-c, ApoA, ApoB, ApoE, Lpa); liver enzymes (ALT, AST, γ-GT, ALP). The secondary outcome is liver fat content by 1H NMR spectroscopy. |
| **Patient Population:** | Patients aged 18 to 65 years with fatty liver (Hepatic fat content assessed by ^1^H MRS were no less than 13%) and impaired glucose metabolism were enrolled. |
| **Inclusion Criteria:** | 1.Patients must have an age range between 18 to 65 years(inclusive).  2. Patients with Liver fat content assessed by 1H MRS≥13%.  3. Patients must meet the criteria for impaired glucose regulation or type 2 diabetes mellitus(FPG≥5.6mmol/L and/or a two hour glucose value≥7.8mmol/L) .Patients with a HbA1c＞7.5% will be excluded from the study.  4.Course of diabetic mellitus less than 1 year |
| **Exclusion Criteria:** | 1.Any causes of chronic liver disease other than NAFLD(such as-but not restricted to- alcohol or drug abuse, medication, chronic hepatitis B or C, autoimmune, etc.);  2. Patients with significantly impaired liver function: ALT or AST≥2 times upper limit of normal;  3. HBsAg(+) and/or HCV-Ab(+), or other liver diseases;  4. Patients with type 1 diabetes mellitus or gestational diabetes or special type diabetes, and patients with BMI＜22Kg/m2;  5. Course of diabetes more than 1 years;  6. Diabetics patients who have taken or are taking oral glucose-lowering drugs or insulin;  7. Diabetics patients with a HbA1c＞7.5% on initial visit;  8. Patients with severe diabetes complications(diabetes ketoacidosis, diabetes coma or with symptomatic of diabetes coma; dysfunction of nerve, retinopathy, dysfunction of kidney);  9. Patients with serum creatinine ≥1.5mg/dL(133umol/L);  10. Patients with a history of cilinically significant heart disease(myocardial infarct, heart failure, and/or severe cardiac rhythm);  11. Complicating severe infection,within 6 months after operation, severe trauma;  12. Patients with excess alcohol consumption≥140g/week(male); ≥70g/week(female);  13. Patients have participated other clinical trials within 24 weeks;  14. Patients with a history of drug allergy to TZDs and berberine;  15. Patients with gestation or possible gestation or lactation, or males or females expecting gestation during clinical trial;  16. Mental diseases patients;  17. Those who refuse to sign informed consent;  18. Any other conditions, which, in the opinion of the investigators would impede compentence or compliance or possiblity of hindering completion of the study;  19. Patients with serum triglyceride≥5.0mmol/L;  20. Patients with thyroid disease, including hyperthyroidism or hypothyroidism. |
| **Randomization:** | The statistical group of Public Health College, Fudan University took responsibility for the preparation and administration of randomization plan. Randomization sequence was generated by SAS8 computer software in a three-center, group randomization manner, and the whole procedure was finished by statistical staff who were not engaged in the study. An envelop randomly labeled with drug assignment was issued only if the database showed that the patient was eligible, had signed the consent statement, and had all required baseline data keyed to the database. The randomization scheme assigned patients to join in one group and accept specific treatment. |
| **Primary Endpoint:** | changes in OGTT (glucose, insulin, AUC of glucose), HbA1c; lipid profile（TC, TG, HDL-c, LDL-c, ApoA, ApoB, ApoE, Lpa); liver enzymes (ALT, AST, γ-GT, ALP). |
| **Study Duration for Each**  **Subject:** | Each subject is followed for 16 weeks; the trial will be completed in 3 year |
| **Number of Centers:** | Three clinical centers in China |
| **Sample Size:** | 180 subjects |
| **Primary Statistical Analysis:** | Statistical analyses will follow the intention-to-treat paradigm, which means that all randomized patients with baseline and 16 week liver enzyme, OGTT, HbA1c,lipid profile and MRS hepatic fat content will be included in the treatment group to which they were assigned. Any randomized patient who does not have the requisite data will be considered unimproved on the primary outcome measure and compared by assigned treatment group. Given the fact that there were two planned primary comparisons, bonferroni-adjusted P values of less than 0.025 were considered to indicate statistical significance. Comparisons of the continuous measures before and after treatment were made with a paired t-test, and the differences from baseline to 16 weeks in BBR plus lifestyle intervention group or PGZ plus lifestyle intervention group were compared with the pure lifestyle intervention group using the analysis of co-variance model respectively, in order to adjust for baseline value. |

1. **STUDY FLOWCHARTS**
   1. **Trial Design**

This study is a three-centers, randomized, open, controlled clinical trial of treatment with pioglitazone, berberine or no drug on the basis of life style intervention for NAFLD patients with IGR or T2DM. The screening period for evaluating eligibility and collecting baseline data lasted up to 4 weeks before randomization. Eligible patients were randomized to receive pioglitazone (15 mg q.d), berberine (0.5g t.i.d.) or no-drug on basis of Life style intervention for 16 weeks. The primary comparison will be made using an intention-to-treat analysis of the change in OGTT (glucose, insulin, AUC of glucose), HbA1c; lipid profile; liver enzymes. The secondary outcome is liver fat content by 1H NMR spectroscopy. A schematic of the trial design is presented in Fig. 1.

**1.2 Schedule of Activities and Assessments**

| Assessment/procedure | **screening（V0）** | **Weeks from randomization** | | | | |
| --- | --- | --- | --- | --- | --- | --- |
|  |  | **V1** | **V2** | **V3** | **V4** | **V5** |
|  | **-4 week** | **0 week** | **4 week** | **8 week** | **12week** | **16week** |
| Consent | ● |  |  |  |  |  |
| Screening questionnaire | ● |  |  |  |  |  |
| Baseline medical history | ● |  |  |  |  |  |
| Follow up medical history | ● | ● | ● | ● | ● | ● |
| Inclusion/exclusion criteria | ● | ● |  |  |  |  |
| Randomization |  | ● |  |  |  |  |
| Health education & diet guide |  | ● | ● | ● | ● | ● |
| Review of concomitant drugs |  |  | ● | ● | ● | ● |
| Review for adverse effects |  |  | ● | ● | ● | ● |
| Drug dispensing |  | ● | ● | ● | ● |  |
| Review of drug adherence |  |  | ● | ● | ● | ● |
| Nutrition questionnaire |  | ● |  |  |  | ● |
| Activity questionnaire |  | ● |  |  |  | ● |
| Physical exam (Detailed/Focused) | D | F | F | F | F | D |
| OGTT with insulin |  | ● |  |  |  | ● |
| Fasting glucose/2h after meal | ● |  | ● | ● | ● |  |
| Fasting lipid profile | ● | ● |  |  |  | ● |
| Liver and renal fuction | ● | ● | ● | ● | ● | ● |
| Blood and urine routine |  | ● | ● | ● | ● | ● |
| HbA1c | ● |  |  |  |  | ● |
| Pregnancy test (females) |  | ● |  |  |  | ● |
| Serum, plasma for banking | ● | ● | ● | ● | ● | ● |
| Hepatic virus anti-body（HBsAb、HCV-Ab） | ● |  |  |  |  |  |
| ECG | ● |  |  |  |  | ● |
| Liver fat content（MRS） | ● | ● |  |  |  | ● |

**2. Background and Rational**

**2.1 The burden of non-alcoholic fatty liver disease and associated metabolic disorders in China**

The global NAFLD prevalence was approximately 20% in 2005 [1,2], and this ratio might increase rapidly with the improvement of human living standard and changes of dietary structure. NAFLD has presented serious threats to human health, not only due to direct damage to hepatocytes, but also hepatic metabolic disorders associated with diabetes and cardiovascular disease(CVD). It was reported that one third of NAFLD patients suffered from metabolic syndrome(MS)[3], and OGTT test showed that 44% NAFLD patients without diabetes history before were abnormal in carbohydrate metabolism, including 13% IGT(impaired glucose tolerance) and 33%DM(diabetes mellitus)[4]. In addition, several studies also showed that NAFLD was an independent risk factor for type 2 diabetes and CVD, and elevation of hepatic enzymes, such as ALT, AST and γ-GT, could increase risks of DM and CVD in people from different ethnic groups [5-12]. To interfere with NAFLD at an early stage had significant benefits to alter its natural history and outcome leading to DM and CVD.

**2.2 Pathogenesis and potential treatments of NAFLD**

Non-alcoholic fatty liver disease (NAFLD) is a common and increasingly recognized liver disease characterized by the accumulation of fat in at least 5% of hepatocytes. The mechanisms underlying NAFLD pathogenesis remain unclear, but prior studies reported that NAFLD was associated with obesity, insulin resistance, dyslipidemia, oxidative stress and abnormal cytokine[13-17]. Histologically, NAFLD can be divided into simple steatosis (non-alcoholic fatty liver: NAFL), non-alcoholic steatohepatitis (NASH) and end-stage liver cirrhosis. Several agents have been found promising as therapy of NAFLD in small clinical trials, but none was approved for general use at present[18-21].

There were no definite therapies for NAFLD currently, due to the uncertainty of its pathogenesis. Lifestyle improvement is a recommended choice, but most patients are unable or unwilling to undertake strict lifestyle control. Anti-obesity drugs (orlistat, rimonabant), antioxidants (vitamin C and E), cytoprotective agents (UDCA), lipid-regulating agents, insulin sensitizers, as well as traditional Chinese medicine have been reported to have beneficial effects on NAFLD.

Several recent pilot studies have shown encouraging results using the insulin sensitizing thiazolidinediones (TZD) to treat NAFLD in non-diabetic individuals [22-25]. Because these studies have had small sample sizes, lacked control groups, or had short follow-up, the reliability of TZD effects on NAFLD still need further evidence from large-scale, randomized, and controlled prospective clinical trial.

Recently, Berberine(BBR) a compound isolated from a Chinese herb, was found to lower serum cholesterol by up-regulating the expression of LDL receptors [26]. Several studies have showed BBR’s potential to correct NAFLD patients’ metabolic disorders and mitigate liver steatosis. However, these studies enrolled only small number of patients and were not placebo-controlled. Furthermore, it was not accurate enough to use ultrasound to evaluate hepatic fat content[27,28].

**3. Study Objective**

**3.1 Primary**

The primary objective of the randomized, three-centers, open, controlled trial is to determine whether pioglitazone or berberine plus lifestyle intervention was superior to pure lifestyle intervention on reducing blood glucose (OGTT glucose, insulin, AUC of glucose and HbA1c), lipid profile（TC, TG, HDL-c, LDL-c, ApoA, ApoB, ApoE, Lpa) and liver enzymes (ALT, AST, γ-GT, ALP) in NAFLD patients with abnormal glucose metabolism.

**3.2 Secondary**

To assess the secondary composite outcomes from the following perspectives:

- Efficacy:
  - Changes in body weight after 16 weeks of treatment.
  - Changes in hepatic fat content by 1H MRS after 16 weeks of treatment (total cholesterol, triglyceride, HDL-C, LDL-C,Apo-A, Apo-B, Apo-E and Lpa) from baseline.
- Safety:
  - Incidence of adverse events.
  - Changes in laboratory parameters (hematology, biochemistry) and vital signs after treatment.

**4. Study design and management overview**

**4.1 Study Design Overview**

The study is a three-centers, randomized, open, controlled clinical trial of treatment with pioglitazone, berberine or no drug on the basis of life style intervention for NAFLD patients with IGR or T2DM. The screening period for evaluating eligibility and collecting baseline data lasted up to 4 weeks before randomization. Eligible patients were randomized to receive pioglitazone (15 mg q.d), berberine (0.5g t.i.d.) or no-drug on basis of Life style intervention for 16 weeks. The primary comparison will be made using an intention-to-treat analysis of the change in OGTT (glucose, insulin, AUC of glucose), HbA1c; lipid profile（TC, TG, HDL-c, LDL-c, ApoA, ApoB, ApoE, Lpa); liver enzymes (ALT, AST, γ-GT, ALP). The secondary outcome is liver fat content by 1H NMR spectroscopy.

**4.2 Study Organization**

**Principal Investigator**

Xin Gao, Zhongshan Hospital, Shanghai, China;

**The executive committee members of the study are as follows:**

Pr. Xin Gao

Department of Endocrinology and Metabolism, Zhongshan Hospital, Fudan University, Shanghai, China

Dr. Hong-Mei Yan

Department of Endocrinology and Metabolism, Zhongshan Hospital, Fudan University, Shanghai, China

Dr. Ming-Feng Xia

Department of Endocrinology and Metabolism, Zhongshan Hospital, Fudan University, Shanghai, China

Pr. Meng-Su Zeng

Department of Radiology, Zhongshan Hospital, Fudan University, Shanghai, China

Dr. Sheng-Xiang Rao

Department of Radiology, Zhongshan Hospital, Fudan University, Shanghai, China

Dr. Xiu-Zhong Yao

Department of Radiology, Zhongshan Hospital, Fudan University, Shanghai, China

Pr. Bai-Shen Pan

Clinical laboratory, Zhongshan Hospital, Fudan University, Shanghai, China

Vice Pr. Wei Deng

School of public health, Fudan University，Shanghai，China

Pr. Wei-Ping Jia

Department of Endocrinology and Metabolism, The sixth people's hospital, Shanghai Jiaotong University, China

Pr. Yun-Qian Bao

Department of Endocrinology and Metabolism, The sixth people's hospital, Shanghai Jiaotong University, China

Dr. Yin-Fang Tu

Department of Endocrinology and Metabolism, The sixth people's hospital, Shanghai Jiaotong University, China

Pr. Jun Liu

Department of Endocrinology and Metabolism, The fifth people's hospital, Fudan University, Shanghai,China

They will convene frequently (teleconferences or physical meetings) to review the status of the trial and available data and will take appropriate actions regarding the conduct of the study.

A face‐to‐face Executive Committee meeting will be organized to make major decisions.

**Safty and monitoring board**

An independent Ethics Committee of Zhongshan Hospital (ECZH) ,Fudan University approved the protocol for the trial and is responsible for monitoring the accumulated interim data as the trial progresses to ensure patient safety and to review efficacy. In addition, the committee is charged with reviewing the quality and timeliness of data collection. Interim data on safety measures requested by the ECZH are reviewed at each of the scheduled semi-annual meetings. Serious adverse events are reviewed by the ECZH as they occur and the ECZH reviews quarterly reports about the count of patients who present hepatic or renal toxicities during the study. One interim efficacy analysis of the primary outcome measure is planned to occur when approximately 50% of the data are complete or when approximately 90 of the 180 patients have completed baseline and 16 week MRS examination. O'Brien-Fleming statistical stopping guidelines for efficacy apply

**4.3 Outcomes**

The primary outcomes are the changes in OGTT (glucose, insulin, AUC of glucose), HbA1c; lipid profile（TC, TG, HDL-c, LDL-c, ApoA, ApoB, ApoE, Lpa); liver enzymes (ALT, AST, γ-GT, ALP). The secondary outcome is liver fat content by 1H NMR spectroscopy.

Safety endpoint includes serious adverse events and non-serous adverse events. Adverse event collection was conducted at each visit after baseline. No specific laboratory safety tests are required during this study. However, if a laboratory abnormality is clinically relevant or leads to an adverse event report, specific appropriate actions may be required. ECG will be performed at the beginning and end of the study.

**4.4 Specimen banking**

Specimens are collected and stored in a central repository for use as approved by the Committee of the trial, endocrine department of Zhongshan Hospital. Specimens include serum, urine and DNA. The blood samples collected at screening visit, and at 4, 8, 12 and 16 week visits are divided into 0.5mL aliquots and stored frozen at −70 °C. Additional blood was collected at the screening visit for extraction of DNA which is stored at −20 °C.

**5. Participant Selection**

During the course of the trial, 180 eligible adults will be identified and recruited from unsolicited referrals to the three participating clinical centers.

**5. 1 Inclusion Criteria**

1.Patients must have an age range between 18 to 65 years(inclusive).

2. Patients with Liver fat content assessed by 1H MRS≥13%.

3. Patients must meet the criteria for impaired glucose regulation or type 2 diabetes mellitus(FPG≥5.6mmol/L and/or a two hour glucose value≥7.8mmol/L) .Patients with a HbA1c＞7.5% will be excluded from the study.

4.Course of diabetic mellitus less than 1 year

5. Informed consent signed

**5. 2 Exclusion Criteria**

1.Any causes of chronic liver disease other than NAFLD(such as-but not restricted to- alcohol or drug abuse, medication, chronic hepatitis B or C, autoimmune, etc.);

2. Patients with significantly impaired liver function: ALT or AST≥2 times upper limit of normal;

3. HBsAg(+) and/or HCV-Ab(+), or other liver diseases;

4. Patients with type 1 diabetes mellitus or gestational diabetes or special type diabetes, and patients with BMI＜22Kg/m2;

5. Course of diabetes more than 1 years;

6. Diabetics patients who have taken or are taking oral glucose-lowering drugs or insulin;

7. Diabetics patients with a HbA1c＞7.5% on initial visit;

8. Patients with severe diabetes complications(diabetes ketoacidosis, diabetes coma or with symptomatic of diabetes coma; dysfunction of nerve, retinopathy, dysfunction of kidney);

9. Patients with serum creatinine ≥1.5mg/dL(133umol/L);

10. Patients with a history of cilinically significant heart disease(myocardial infarct, heart failure, and/or severe cardiac rhythm);

11. Complicating severe infection,within 6 months after operation, severe trauma;

12. Patients with excess alcohol consumption≥140g/week(male); ≥70g/week(female);

13. Patients have participated other clinical trials within 24 weeks;

14. Patients with a history of drug allergy to TZDs and berberine;

15. Patients with gestation or possible gestation or lactation, or males or females expecting gestation during clinical trial;

16. Mental diseases patients;

17. Those who refuse to sign informed consent;

18. Any other conditions, which, in the opinion of the investigators would impede compentence or compliance or possiblity of hindering completion of the study;

19. Patients with serum triglyceride≥5.0mmol/L;

20. Patients with thyroid disease, including hyperthyroidism or hypothyroidism.

**6. Treatments**

Patients who signed an informed consent statement and who met the eligibility criteria were randomly assigned to one of three groups for 16 weeks of treatment:

Group 1: Pioglitazone (15 mg q.d.) and Life style intervention

Group 2: Berberine (0.5g, t.i.d.) and life style intervention

Group 3: Life style intervention only without drugs

Pioglitazone (Actlns®, Taiyang Pharmaceuticals Beijing, China, Inc.) is administered as a single tablet of 15 mg per day orally 30 minutes before the morning meal. The rationale for choosing this dosage was based upon earlier pilot studies that examined the safety and efficacy of pioglitazone in patients with NAFLD[22-25].

Berberine ( Berberine®, Huashi Pharmaceuticals Shanghai, China, Inc.) is administered three times a day, at a dosage of 0.5g orally each time. The berberine dose chosen for this trial (0.5g t.i.d) is more than the dose recommended clinically, but still within the range of safety that has been tested in previous studies[27,28].

In addition to study medications, participants receive standardized recommendations concerning life-style modification (dietary modification, weight loss, exercise), use of prescription or non-prescription medicines or herbal remedies or dietary supplements, consumption of alcohol, and management of various co-morbid illnesses. All participants follow calorie limited diet that subtracts 500kcal from daily mean calorie intake before entering the treatment (daily mean calorie intake is calculated through diet diary during screening period) and are required to keep daily food diaries. We also provide participants with general recommendations to achieve more than 150 min medium intensity aerobic exercise per week with heart rate around 50-70% maximum, or more than 90 minutes higher-intensity aerobic exercise per week with heart rate around 70% maximum.

**7. Study Drug Handling**

**7.1 Supply and Storage**

The drugs used in the trial, pioglitazone and berberine, were purchased from the pharmceutical companies to ensure the same lot number. All investigational drug supplies in the study will be stored in a secure, safe place, under the responsibility of the Investigator or other authorized individual, and under the conditions described on the labeling.

**7.2 Dispense and management**

The investigator should dispense the specific drugs determined at randomization to eligible participants at each visit from V0 to V4 and count the tablets returned by the participant. It is the investigator’s responsiblility to ensure that an accurate record of invesigational product issued and retured is maintained.

**7.3 Prohibited Concomitant Treatment**

Participants are not allowed any prescription or over-the-counter medication or herbal remedy to improve NAFLD or IGR/T2DM during screening and treatment phase of the trial. The anti-NAFLD agents are defined as lipid-regulating drugs(statins, fibrates), silybin, ursodeoxycholic acid, Polyene Phosphatidylcholine, Vitamin E, and some herbs regulating lipid and protecting liver function. The anti-IGR or T2DM includes insulin, insulin sensitizer (Rosiglitazone), sulfonylureas, biguanides, α-glycosidase inhibitors.

**7.4 Treatment Accountability and Compliance**

Compliance will be assessed by counting returned tablets at each visit. The investigator (or delegates) will complete the appropriate page of the CRF or study drug inventory log form. The date of study drug interruption must also be recorded.

**7.5 Treatment Discontinuation**

The investigational product (IP) should be continued whenever possible. If the IP is stopped, it should be determined if the discontinuation can be made temporarily; permanent IP discontinuation should be a last resort. Any IP discontinuation should be fully documented in the CRF. In any case, the patient should remain in the study as long as possible.

Pregnancy will lead to definitive treatment discontinuation in all cases.

**7.5.1 Temporary treatment discontinuation with investigational product**

Re-initiation of treatment with the Investigational Product will be done under close and appropriate clinical/and or laboratory monitoring once the Investigator has considered according to his/her best medical judgment that the role of the Investigational Product(s) in the occurrence of the event concerned was unlikely and there is no other contraindication to continuing in the study.

All temporary treatment discontinuation (< 10 days) and the date of treatment re-initiation should be recorded by the Investigator on the appropriate CRF pages when considered to be confirmed

**7.5.2 Definition treatment discontinuation with investigational product(s)**

A patient should discontinue the study drug for any of the following reasons:

- Intercurrent condition that requires discontinuation of the study (e.g. lab abnormalities).
- Positive serum pregnancy test or desire to become pregnant
- Contraception cessation

**7.5.3 Handling of patients after definitive treatment discontinuation**

Patients will be followed according to the study procedures as specified in this protocol up to the scheduled date of study completion, or up to recovery or stabilization of an AE, whichever comes last.

All withdrawals should be recorded by the Investigator on the appropriate CRF pages when considered to be confirmed.

All patients who have discontinued the study drug prior to the last visit should have a complete end-of-study visit at three months.

For patients considered lost to follow-up, the CRF must be completed up to the last visit performed. The Investigator should make every effort to contact the patient and to identify the reason why he/she failed to attend the visit and to determine his/her health status.

**8. Safety**

**8.1 Adverse event monitoring**

All events will be managed and reported in compliance with all applicable regulations and will be included in the final Clinical Study Report.

**8.2 Definition of adverse event and serious adverse event**

An Adverse Event is any untoward medical occurrence in a patient or clinical investigation patient administered a pharmaceutical product and which does not necessarily have to have a causal relationship with this treatment. Adverse events include symptoms (nausea, chest pain, et al.), physical signs (tachycardia, hepatomegaly ) or abnormal laboratory examination results.

A Serious Adverse Event is any untoward medical occurrence that at any dose:

 Results in death, or

 Is life-threatening, or

Requires inpatient hospitalization or prolongation of existing hospitalization, or

Results in persistent or significant disability/incapacity, or

 Is a congenital anomaly/birth defect, or

 Is a medically important event

Note: The term "life-threatening" in the definition of "serious" refers to an event in which the patient was at risk of death at the time of the event; it does not refer to an event which hypothetically might have caused death if it were more severe.

Medical and scientific judgment should be exercised in deciding whether expedited reporting is appropriate in other situations, such as important medical events that may not be immediately life-threatening or result in death or hospitalization but may jeopardize the patient or may require intervention to prevent one of the other outcomes listed in the definition above.

A non-serious adverse event (NSAE) is any event that does not fit in the definition of SAE. Again, it is important to collect information on all NSAEs whether or not it is related to the study event.

The intensity of the NSAE was classified as the follows:

1=Mild: Transient or mild discomfort; no medical intervention/therapy required.

2=Moderate: Mild to moderate limitation in activity - some assistance may be needed; no or minimal medical intervention/therapy required.

3=Severe: Marked limitation in activity, some assistance usually required; medical intervention/therapy required, hospitalization possible.

The NSAEs are classified into six grades according to their causal relationship with the treatment.

1. Sure
2. Very probable
3. Probable
4. Possible
5. Doubtful
6. Appears excluded

**8.3 Adverse event recording**

All Adverse Events regardless of seriousness or relationship to Investigational Product, spanning the time from the first visit planned in the Clinical Trial Protocol/signature of the informed consent (i.e., occurring during the washout period) to the last visit planned in the

protocol, the relative treatment are to be recorded on the corresponding page(s) included in the Case Report Form. Whenever possible, symptoms should be grouped as a single syndrome or diagnosis. The Investigator should specify the date of onset, date of cease, intensity, action taken with respect to Investigational Product, corrective treatment/therapy given, outcome and his/her opinion as to whether there is a reasonable possibility that the Adverse Event was caused by the invesigational product.

All adverse events during the study period must be recorded, Efficacy endpoints will not be considered as AEs except if, because of the course or severity or any other features of such events, the Investigator, according to his/her best medical judgment, considers these events as exceptional in this medical condition.

**8.4 Adverse event treatment**

In the case of a Serious Adverse Event the Investigator must immediately:

SEND (within 1 working day, preferably by fax) the signed and dated corresponding page(s) in the Case Report Form to the representative of the Monitoring Team whose name, address and fax number appear on the Clinical Trial Protocol;

ATTACH a photocopy of all examinations carried out and the dates on which these examinations were performed. Care should be taken to ensure that the patient's identity is protected and the patient's identifiers in the Clinical Trial are properly noted on any copy of source documents provided to the Sponsor. For laboratory results, include the laboratory normal ranges;

Follow‐up of any Serious Adverse Event that is fatal or life threatening should be provided within one additional calendar week.

**8.5 Follow-up**

The Investigator should take all appropriate measures to ensure the safety of the patients; notably, he should follow up the outcome of any Adverse Events (clinical signs, laboratory values or other, etc.) until the return to normal or stabilization of the patient's condition;

In the case of any Serious Adverse Event, the patient must be followed up until clinical recovery is complete and laboratory results have returned to normal, or until progression has been stabilized. This may imply that follow‐up will continue after the patient has left the Clinical Trial and that additional investigations may be requested by the Monitoring Team;

In the case of any Serious Adverse Event brought to the attention of the Investigator at any time after cessation of Investigational Product and considered.

**9 Study Procedures**

**9.1 Screening (V0): 4 weeks before randomization**

A screening form will be completed for all patients in whom enrollment is considered. The patient will receive complete information about the study, both orally and in writing. Consent will be obtained directly from the subject prior to enrollment. Baseline evaluation will include patient demographic information, medications, past medical history, family history, cigarette and alcohol use, examination findings. A physical examination will be performed, including measurement of weight (kg), waist and hip circumference, and vital signs (supine systolic and diastolic blood pressure, heart rate). Laboratory tests will be performed (liver and renal function, OGTT blood glucose, HbA1c, HbsAg, HCV-Ab, electrocardiogram and hepatic fat content by 1H MRS).

**9.2 Visit 1 (V1)**

Evaluation before randomization will include patient demographic information, medications, diet and exercise information, past medical history, family history, cigarette and alcohol use, and physical examination. Laboratory tests will be performed (liver and renal function, OGTT blood glucose + insulin, HbA1c, lipid profile, blood and urine routine, urine pregnancy test and hepatic fat content by 1H MRS).

The statistician will develop a master randomization list, with stratification blocked to assure an equal distribution of treatments at each participating site. A study number will correspond to a specific medication packet. This identifying number will be coded on the enrollment confirmation.

The patient will receive 4-week study medication and be instructed how to take the study medication. Time of first study drug administration will be recorded. Study drug will be started the next day after randomization. participants receive standardized recommendations concerning life-style modification (dietary modification, weight loss, exercise).

 An appointment will be made for next study visit (4 weeks after randomization).

**9.3 Visit 2 (V2): 4 weeks after randomization**

Evaluation will include patient concomitant diseases and Concomitant medications, diet and exercise diary. A physical examination will be performed, including measurement of weight (kg) , waist and hip circumference, and vital signs (supine systolic and diastolic blood pressure, heart rate)Laboratory tests will be performed (liver and renal function, OGTT blood glucose + insulin, blood and urine routine).

participants receive standardized recommendations concerning life-style modification (dietary modification, weight loss, exercise).

Compliance will be assessed by counting returned tablets using the equation as the follows:

Compliance=(dispensed drug tablets- returned drug tablets) /planned drug tablets

The compliance should be been 80%-120%.

All adverse events will be managed and reported in compliance with all applicable regulations.

The investigator should dispense the specific drugs determined at randomization to eligible participants at each visit.

An appointment will be made for next study visit (8 weeks after randomization).

**9.4 Visit 3 (V3): 8 weeks after randomization**

Evaluation will include patient concomitant diseases and Concomitant medications, diet and exercise diary. A physical examination will be performed, including measurement of weight (kg) , waist and hip circumference, and vital signs (supine systolic and diastolic blood pressure, heart rate). Laboratory tests will be performed (liver and renal function, OGTT blood glucose + insulin, blood and urine routine).

participants receive standardized recommendations concerning life-style modification (dietary modification, weight loss, exercise).

Compliance will be assessed by counting returned tablets using the equation as the follows:

Compliance=(dispensed drug tablets- returned drug tablets) /planned drug tablets

The compliance should be been 80%-120%.

All adverse events will be managed and reported in compliance with all applicable regulations.

The investigator should dispense the specific drugs determined at randomization to eligible participants at each visit.

An appointment will be made for next study visit (12 weeks after randomization).

**9.5 Visit 4 (V4): 12 weeks after randomization**

Evaluation will include patient concomitant diseases and Concomitant medications, diet and exercise diary. A physical examination will be performed, including measurement of weight (kg) , waist and hip circumference, and vital signs (supine systolic and diastolic blood pressure, heart rate). Laboratory tests will be performed (liver and renal function, OGTT blood glucose + insulin, blood and urine routine).

participants receive standardized recommendations concerning life-style modification (dietary modification, weight loss, exercise).

Compliance will be assessed by counting returned tablets using the equation as the follows:

Compliance=(dispensed drug tablets- returned drug tablets) /planned drug tablets

The compliance should be been 80%-120%.

All adverse events will be managed and reported in compliance with all applicable regulations.

The investigator should dispense the specific drugs determined at randomization to eligible participants at each visit.

An appointment will be made for next study visit (16 weeks after randomization).

**9.6 Visit 5 (V5): 16 weeks after randomization**

Evaluation will include patient concomitant diseases and Concomitant medications, diet and exercise diary. A physical examination will be performed, including measurement of weight (kg) , waist and hip circumference, and vital signs (supine systolic and diastolic blood pressure, heart rate). Laboratory and imaging tests will be performed (liver and renal function, OGTT blood glucose + insulin, HbA1c, blood and urine routine, lipid profile, urine pregancy test, electrocardiogram and hepatic fat content by 1H MRS).

Compliance will be assessed by counting returned tablets using the equation as the follows:

Compliance=(dispensed drug tablets- returned drug tablets) /planned drug tablets

The compliance should be been 80%-120%.

All adverse events will be managed and reported in compliance with all applicable regulations.

An appointment will be made if additional follow up is necessary. Conditions which need additional follow up include adverse event or abnormal laboratory test result.

**10 Statistical Considerations**

**10.1 Statistical Analysis Plans**

This section is an overview of the statistical considerations. Complete details can be found in the Statistical Analysis Plan (SAP). It provides the general specifications for the analysis of the

data to be collected and presented in the Clinical Study Report. A final SAP will be issued prior to database lock and before code breaking. The SAP will define all “pre-specified, planned analyses.”

**10.2 Sample Size Estimation**

The planned sample size for the trial was 180 patients with equal allocation to each of the three treatment groups (60 per group). The sample size estimates were based upon a two-group, binomial comparison of the proportions of patients satisfying the decrease in liver fat content over the course of treatment either with pioglitazone or berberine. Since our current study had three treatment groups and two primary hypotheses, the assumption was made that the two primary comparisons, pioglitazone vs. no-drug control and berberine vs. no-drug control, required the type I error estimate be reduced from 0.05 to 0.016 in accordance with three-group comparison. Expected proportions improved were speculated using pilot data from pioglitazone-treat NAFLD studies. There were no available data at the time of study design to estimate liver steatosis response with berberine, which was assumed, for purposes of planning the trial, to be the same as for pioglitazone.

The sample size calculations were performed using the formula: n1=n2=2[(tα+ t2β)s/δ]^2^ with a standard deviation of NAFLD patients liver fat content (s=6.87%, from our prior study), with an expected difference in proportion of improvement between pioglitazone or berberine group and the control group (δ=0.05), with an α level of two-sided type I error (0.016, corrected for three-group comparison), and with a β level of type II error (0.10; i.e., 90% power). The number per group, using the above assumptions was 52. Inflating this number by the 15% expected missing data rate yielded approximately 60 patients per group, or a total of 180 for the trial.

**10.3 Statistical Analyses**

Statistical analyses for the two primary hypotheses will follow the intention-to-treat paradigm, which means that all randomized patients with baseline and 16 week liver enzyme, OGTT, HbA1c,lipid profile and 1H MRS hepatic fat content will be included in the treatment group to which they were assigned. Missing values will remain missing and patients will be censored at their last follow‐up assessment.

The chi-square test and analysis of covariance or Kruskal-Wallis H test were used to compare baseline variables among the groups for categorical and continuous variables, respectively. Comparisons of the continuous measures before and after treatment were made with the use of a paired t-test, and the differences from baseline to 16 weeks in BBR plus lifestyle intervention group or pioglitazone plus lifestyle intervention group were compared with the lifestyle intervention group using the analysis of co-variance model respectively, in order to adjust for baseline value. Given the fact that there were two planned primary comparisons, bonferroni-adjusted P values of less than 0.025 were considered to indicate statistical significance.

**11 Ethical and Regulatory Standards**

**11.1 Ethical Principles**

This Clinical Trial will be conducted in accordance with the principles laid down by the 18^th^ World Medical Assembly (Helsinki, 1964), and approved by an independent Ethics Committee of Zhongshan Hospital (ECZH), Fudan University, and all subjects gave written informed consent.

**11.2 Laws and regulations**

This Clinical Trial will be conducted in compliance with all international laws and regulations, and Chinese laws and regulations in which the Clinical Trial is performed, as well as any applicable guidelines.

**11.3 Informed Consent**

The Investigator (according to applicable regulatory requirements), or a person designated by the Investigator and under the Investigator's responsibility, should fully inform the Patient of all pertinent aspects of the Clinical Trial including the written information giving approval/favorable opinion by the Ethics Committee of Zhongshan Hospital (ECZH), Fudan University. All participants should be

informed to the fullest extent possible about the study, in language and terms they are able to understand.

Prior to a patient's participation in the Clinical Trial, the written Informed Consent Form should be signed, name filled in and personally dated by the patient or by the patient's legally acceptable representative, and by the person who conducted the informed consent discussion. A

copy of the signed and dated written Informed Consent Form will be provided to the patient.

**References**

Centro Studi Fegato, AREA Science Park, Basovizza etal. Prevalence of and risk factors for nonalcoholic fatty liver disease: the Dionysos nutrition and liver study. Hepatology. 2005 Jul;42(1):44-52.

2 Leon A. Adams, Paul Angulo, Keith D et al. Nonalcoholic fatty liver disease. CMAJ. 2005; 172(7): 899 -905.

3 Marchesini G, Bugianesi E, Forlani G, et al. Nonalcoholic fatty liver, steatohepatitis, and the metabolic syndrome. Hepatology.2003;37: 917-23.

4 Sargin, Mehmet; Uygur–Bayramiçli, Oya; Sargin, Halu, et al. Association of Nonalcoholic Fatty Liver Disease With Insulin Resistance: Is OGTT Indicated in Nonalcoholic Fatty Liver Disease? J Clin Gastroenterol. 2003;37(5):399-402.

5 Vozarova B, Stefan N, Lindsay RS, et al. High alanine aminotransferase is associated with decreased hepatic insulin sensitivity and predicts the development of type 2 diabetes. Diabetes.2002,51: 1889 -1895

6 Hanley AJ, Williams K, Festa A, et al. Insulin resistance atherosclerosis study. Elevations in markers of liver injury and risk of type 2 diabetes: the insulin resistance atherosclerosis study. Diabetes. 2004,53:2623-2632.

7 Perry IJ, Wannamethee SG, Shaper AG. Prospective study of serum gamma-glutamyltransferase and risk of NIDDM. Diabetes Care. 1998,21:732-737.

8 Lee DH, Ha MH, Kim JH, et al. Gamma-glutamyltransferase and diabetes–a 4 year follow-up study. Diabetologia. 2003,46:359-364.

9 Lee DH, Jacobs DR Jr, Gross M, et al. Gamma-glutamyltransferase is a predictor of incident diabetes and hypertension: the Coronary Artery Risk Development in Young Adults (CARDIA) Study. Clin Chem. 2003,49:1358-1366.

10 Lee DH, Silventoinen K, Jacobs DR Jr, et al. Gamma-Glutamyltransferase, obesity, and the risk of type 2 diabetes: observational cohort study among 20,158 middle-aged men and women. .J Clin Endocrinol Metab. 2004,89: 5410-5414..

11 Sattar N, McConnachie A, Ford I, et al. Serial metabolic measurements and conversion to type 2 diabetes in the west of Scotland coronary prevention study: specific elevations in alanine aminotransferase and triglycerides suggest hepatic fat accumulation as a potential contributing factor. Diabetes.2007,56: 984-991.

12 Schindhelm RK, Dekker JM, Nijpels G, et al. Alanine aminotransferase predicts coronary heart disease events: A 10-year follow-up of the Hoorn Study. Atherosclerosis.2007,191:391-396.

13 Marchesini G, Brizi M, Morselli-Labate AM, et al. Association of nonalcoholic fatty liver disease with insulin resistance. Am J Med 1999;107:450–5.

14 Pagano G, Pacini G, Musso G, et al. Nonalcoholic steatohepatitis, insulin resistance, and metabolic syndrome: further evidence for an etiologic association. Hepatology 2002;35:367–72.

15 Weltman MD, Farrell GC, Hall P, Ingelman-Sundberg M, Liddle C. Hepatic cytochrome P450 2E1 is increased in patients with nonalcoholic steatohepatitis. Hepatology 1998;27:128–33.

16 Chalasani N, Deeg MA, Crabb DW. Systemic levels of lipid peroxidation and its metabolic and dietary correlates in patients with nonalcoholic steatohepatitis. Am J Gastroenterol 2004;99:1497–502.

17 Tilg H, Diehl AM. Cytokines in alcoholic and nonalcoholic steatohepatitis. New Engl J Med 2000;343:1467–76.

18 Lirussi F, Azzalini L, Orando S,et al. Antioxidant supplements for non-alcoholic fatty liver disease and/or steatohepatitis. Cochrane Database Syst Rev. 2007,24;(1):CD004996.

19 Angelico F, Burattin M, Alessandri C,et al. Drugs improving insulin resistance for non-alcoholic fatty liver disease and/or non-alcoholic steatohepatitis. Cochrane Database Syst Rev. 2007,24;(1):CD005166

20 Lirussi F, Mastropasqua E, Orando S,et al. Probiotics for non-alcoholic fatty liver disease and/or steatohepatitis. Cochrane Database Syst Rev. 2007,24;(1):CD005165.

21 Orlando R, Azzalini L, Orando S,et al. Bile acids for non-alcoholic fatty liver disease and/or steatohepatitis. Cochrane Database Syst Rev. 2007,24;(1):CD005160.

22 Belfort R, Harrison SA, Brown K,et al. A placebo-controlled trial of pioglitazone in subjects with nonalcoholic steatohepatitis. N Engl J Med. 2006;355(22):2297-307.

23 Lutchman G, Promrat K, Kleiner DE,et al. Changes in serum adipokine levels during pioglitazone treatment for nonalcoholic steatohepatitis: relationship to histological improvement. Clin Gastroenterol Hepatol. 2006;4(8):1048-52.

24 Sanyal AJ, Mofrad PS, Contos MJ,et al. A pilot study of vitamin E versus vitamin E and pioglitazone for the treatment of nonalcoholic steatohepatitis. Clin Gastroenterol Hepatol. 2004;2(12):1107-15.

25 Promrat K, Lutchman G, Uwaifo GI,et al. A pilot study of pioglitazone treatment for nonalcoholic steatohepatitis. Hepatology. 2004;39(1):188-96.

26 Kong W, Wei J, Abidi P, et al. Berberine is a novel cholesterol-lowering drug working through a unique mechanism distinct from statins.Nat Med. 2004,10(12):1344-51.

27魏敬, 吴锦丹, 蒋建东等. 盐酸小檗碱治疗2型糖尿病合并脂肪肝的临床研究.中西医结合肝病杂志,2004,14(6):334-336.

28刘才乐,张少君,罗禅清等.盐酸小聚碱治疗非酒精性脂肪肝34例.实用医学杂志, 2006, 22(21): 2519 -2520.
