# Supplementary material for: Efficacy of Berberine in Patients with Non-Alcoholic Fatty Liver Disease
Source: PLoS One. 2015 Aug 7;10(8):e0134172. doi: 10.1371/journal.pone.0134172 (PMC4529214; doi:10.1371/journal.pone.0134172)
Supplement: S2 Protocol — (DOC) [file pone.0134172.s005.doc]

**盐酸吡格列酮及盐酸小檗碱治疗伴糖代谢异常的**

**非酒精性脂肪肝性肝病随机对照开放临床试验**

| 项目负责人： | 高 鑫 |
| --- | --- |
| (主要研究者) |  |
| 项目来源： | 07上海市科委创新行动计划 |
| 项目名称： | 糖脂代谢疾病的发病机理及防治的基础研究 |
| 项目编号： | 07JC14011 |

复旦大学附属中山医院内分泌科

第 4 版 修订日期：2008-8-8

**方案修订记录**

| 方案版本 | 人员 / 单位 | 日期 |
| --- | --- | --- |
| 第1版 | 高鑫 颜红梅/复旦大学附属中山医院内分泌科 | 2007年10月22日 |
| 第2版 | 高鑫 颜红梅 邓伟 金丕焕/复旦大学附属中山医院内分泌科 复旦大学公共卫生学院统计教研室 | 2007年12月11日 |
| 第3版 | 高鑫 颜红梅 邓伟 金丕焕/复旦大学附属中山医院内分泌科 复旦大学公共卫生学院统计教研室 | 2008年04月16日 |
| 第4版 | 高鑫 刘蒙 贾伟平 包玉倩/复旦大学附属中山医院内分泌科 上海交通大学附属第六人民医院内分泌科 | 2008年08月08日 |

GLOSSARY

| ALT | 丙氨酸氨基转移酶 |
| --- | --- |
| ApoA | 载脂蛋白A |
| ApoB | 载脂蛋白B |
| ApoE | 载脂蛋白E |
| AST | 天冬氨酸氨基转移酶 |
| BMI | 体重指数 |
| BRHS | 英国地区心脏研究 |
| CHD | 冠心病 |
| CVD | 心血管疾病 |
| DM | 糖尿病 |
| DRQ | 疑问解答表 |
| ECG | 心电图 |
| FAS | 全分析集 |
| HBsAg | 乙型肝炎病毒表面抗原 |
| HbA1c | 糖化血红蛋白 |
| HCV-Ab | 丙型肝炎病毒抗体 |
| HDL-c | 高密度脂蛋白胆固醇 |
| ^1^H MRS | 质子磁共振波谱 |
| IGT | 糖耐量异常 |
| IRAS | 胰岛素抵抗动脉硬化研究 |
| ITT | 意向性治疗 |
| LDL-c | 低密度脂蛋白胆固醇 |
| Lpa | 脂蛋白a |
| MS | 代谢综合征 |
| NAFLD | 非酒精性脂肪性肝病 |
| NASH | 非酒精性脂肪性肝炎 |
| NIDDK | 美国国家糖尿病、消化与肾脏病研究院 |
| NSAE | 非严重不良事件 |
| OGTT | 口服葡萄糖耐量试验 |
| PIVENS | 吡格列酮 vs维生素E vs安慰剂治疗  非糖尿病的非酒精性脂肪性肝炎Ⅲ期临床研究 |
| PPARγ | 过氧化物酶体增殖体激活受体γ |
| PPS | 符合方案分析集 |
| γ-GT | γ-谷氨酰氨基转移酶 |
| SAE | 严重不良事件 |
| SS | 安全集 |
| TC | 总胆固醇 |
| TG | 甘油三酯 |
| WOSCOP | 苏格兰西部冠脉疾病预防研究 |

目 录

| 一、  研究背景 | 第6页 |
| --- | --- |
| 二、  研究目的 | 第8页 |
| 三、受试者条件 | 第8页 |
| 3.1 入选标准 | 第8页 |
| 3.2 排除标准 | 第9页 |
| 3.3 剔除标准 | 第9页 |
| 四、干预方法与研究药物 | 第9页 |
| 4.1 单纯生活方式干预： | 第10页 |
| 4.2 选择干预药物的依据 | 第10页 |
| 五、治疗方法 | 第11页 |
| 六、合并用药 | 第11页 |
| 七、 试验药品的管理 | 第11页 |
| 7.1 试验药物的提供和储存 | 第11页 |
| 7.2 试验药物的分发和管理 | 第12页 |
| 八、研究过程 | 第13页 |
| 8.1 流程图 | 第13页 |
| 8.2 试验过程与观察项目 | 第14页 |
| 8.3 研究路线 | 第16页 |
| 九、病例数量与安排 | 第16页 |
| 9.1 样本量计算依据： | 第16页 |
| 9.2病例安排： | 第17页 |
| 十、临床观察项目和指标 | 第17页 |
| 10.1确定的检测指标 | 第17页 |
| 10.2研究终点 | 第17页 |
| 十一、非严重不良事件（NSAE）的观察 | 第17页 |
| 11.1非严重不良事件的定义 | 第17页 |
| 11.2非严重不良事件的记录 | 第18页 |
| 11.3非严重不良事件的严重程度分级 | 第18页 |
| 11.4非严重不良事件与研究药物关系的判断标准 | 第18页 |
| 11.5 本试验非严重不良事件的处理 | 第19页 |
| 十二、严重不良事件（SAE） | 第19页 |
| 12.1严重不良事件的判定 | 第19页 |
| 12.2 严重不良事件的处理 | 第19页 |
| 十三、随机化方法 | 第20页 |
| 十四、数据管理 | 第20页 |
| 十五、统计分析 | 第21页 |
| 15.1 分析人群和分析工具 | 第21页 |
| 15.2疗效评价 | 第21页 |
| 15.3安全性评价 | 第22页 |
| 15.4 其他分析 | 第23页 |
| 十六、伦理要求 | 第23页 |
| 十七、资料保存 | 第23页 |
| 十八、总结 | 第23页 |
| 十九、时间安排 | 第24页 |
| 二十、其他事宜 | 第24页 |
| 二十一、组织领导结构 | 第24页 |
| 参考文献 | 第25页 |
| 附录1：知情同意书 | 第27页 |

1. **研究背景**

随着生活水平的提高和饮食结构的改变，非酒精性脂肪性肝病（NAFLD）的患病率逐渐上升，其全球平均患病率为20% [[[1]](#endnote-2),[[2]](#endnote-3)]，上海市区成年人中患病率为15.35%[[[3]](#endnote-4)]。该病包括单纯性肝脂肪变性、非酒精性脂肪性肝炎（NASH，需肝穿刺明确诊断），以及终末期的肝硬化。部分患者可演变为肝细胞癌[[[4]](#endnote-5)]。

脂肪肝对机体的危害，除了增加肝病相关的发病率与死亡率，更重要的是引起代谢异常性疾病，从而引起糖尿病与心血管疾病的发病率与死亡率的增加[[[5]](#endnote-6)]。NAFLD人群是代谢综合征（MS）、2型糖尿病（DM）、心血管疾病（CVD）的“后备军”。国外研究报道，NAFLD患者中约1/3伴有MS[[[6]](#endnote-7)]，无糖尿病史的NAFLD患者经OGTT试验，44％存在糖代谢异常，其中13%为IGT，约33%为DM[[[7]](#endnote-8)]；我课题组最近报道：在上海无糖尿病史的NAFLD人群中，31.4%为MS，糖代谢异常者高达43.2%，其中新诊断糖尿病者为14.4%[[[8]](#endnote-9)]，与国外研究结果相似。国外前瞻性研究发现脂肪肝可以预测2型糖尿病和CVD的发生。分析12个前瞻性研究中有9个研究结果发现：肝酶增高是预测2型糖尿病的独立危险因素（白种人中常用肝酶增高作为肝脏脂肪变性的指标）。对正常糖耐量的Pima印地安人平均随访6.9年，在随访的任何时点，ALT处于上四分位水平(≥59U/L)者，糖尿病累积发病率均高于ALT处于下四分位水平(≥21U/L)者 [[[9]](#endnote-10)]；在IRAS研究中（Insulin Resistance Atherosclerosis Study），AST和ALT的升高均是独立于BMI的2型糖尿病患病风险因素[[[10]](#endnote-11)]；BRHS(British Regional Heart Study)研究对7458例非糖尿病男性随访12.8年的结果显示：血浆γ-GT是独立于BMI的2型糖尿病患病风险因素[[[11]](#endnote-12)]；另外，韩国[[[12]](#endnote-13)]、美国黑人、白人[[[13]](#endnote-14)]、芬兰[[[14]](#endnote-15)]人群的研究和最近WOSCOP研究[[[15]](#endnote-16)]均报道了类似的结果。荷兰Hoorn Study研究显示基线血浆ALT升高增加了受试者冠心病（CHD）风险，而且独立于所有其他CHD危险因素[[[16]](#endnote-17)]。一项对132例NAFLD患者随访18年结果显示，心血管疾病（CVD）是该人群第二大死因[[[17]](#endnote-18)]。另一项对420例NAFLD患者平均随访7.6年，总死亡率高于普通人群，主要死因之一是CVD [[[18]](#endnote-19)]。这些研究结果说明两个问题：1) 提示肝脏脂肪含量增高先于2型糖尿病和心血管疾病的发生，强烈提示肝脏脂肪含量增加在2型糖尿病和心血管疾病的发病机制中起着关键性的作用。2) NAFLD患者是2型糖尿病和CVD及动脉粥样硬化疾病的“后备军”，为了防治“后备军”向终末疾病进展，必须将防治中心重点前移，因此加强NAFLD群体的防治研究、探索新的有效的干预措施是有效防治2型糖尿病与CVD的迫切任务。另外，脂肪肝也是血肝酶升高的主要原因之一，对于青中年人群，肝酶的升高对患者工作与生活造成了很大不便，尽快降低肝酶，减轻肝脏脂肪积聚可减轻脂肪肝患者心理压力。

诊断和评价NAFLD疗效的金标准是肝穿刺病理诊断方法，肝脏脂肪含量>5%定义为脂肪肝。但这一方法的有创性限制了其在临床及人群研究中的广泛应用。影像学检查如普通B超，是非定量研究，对低于30%的肝脏脂肪变性的检测不敏感，一般仅用于对脂肪肝定性诊断；CT半定量方法较普通B超诊断进了一步，但是仍然不能精确定量。评价方法的缺陷一度成为限制NAFLD基础与临床研究的“瓶颈”。国外自1994年开始应用质子磁共振波谱（^1^H MRS）无创性精确测定肝脏及骨骼肌脂肪含量的方法[[[19]](#endnote-20)]，被称作无创测量的“金标准”，使得NAFLD的研究取得了长足的进展，检索近3年发表在国际权威杂志上有关NAFLD的文章均采用此定量方法。2006年在上海市科委（06ZR14020）的支持下，我课题组在国内率先建立了^1^H MRS测定肝脏脂肪含量的方法[[[20]](#endnote-21)]，结果显示^1^H MRS测定肝脏脂肪含量方法精确稳定，与肝穿刺测定的肝脏脂肪含量相关性很高（相关系数 *r*=0.814）。^1^H MRS方法的建立为我国NAFLD的基础与临床研究提供了稳定可靠的平台，缩小了我们与国际同类研究的差距。

由于NAFLD的病因及发病机制不明，目前对其治疗方法缺乏病因治疗的依据。已经报道的干预研究，包括运动减肥，饮食限制方案、噻唑烷二酮类等药物治疗，均为小样本研究，部分研究通过肝穿刺评价治疗前后的疗效，部分研究通过磁共振波谱分析评估疗效，迄今还缺乏大样本、随机对照的前瞻性干预研究的证据，尚无公认的药物治疗方法[[[21]](#endnote-22),[[22]](#endnote-23),[[23]](#endnote-24),[[24]](#endnote-25)]。

吡格列酮为噻唑烷二酮类口服抗糖尿病药，为过氧化物酶体增殖体激活受体γ(PPARγ)的激动剂，通过提高外周和肝脏的胰岛素敏感性而控制血糖水平。其主要作用机理为激活脂肪、骨骼肌和肝脏等胰岛素作用组织的PPARγ核受体，从而调节胰岛素应答基因的转录，控制血糖的生成、转运和利用。国外有关吡格列酮治疗NAFLD临床试验的文献已经发表的有4篇[[[25]](#endnote-26), [[26]](#endnote-27), [[27]](#endnote-28), [[28]](#endnote-29) ]，总样本数在18－55例之间，均为小规模研究，结果提示吡格列酮可以明显减少NAFLD患者肝脏脂肪含量；改善非酒精性脂肪性肝炎患者的生物学指标及肝脏组织学^[25,27^,[[29]](#endnote-30)]。国内有学者应用吡格列酮对伴有2型糖尿病的NAFLD患者进行治疗[[[30]](#endnote-31)]，结果示吡格列酮治疗组在改善脂肪肝程度、降低空腹胰岛素及改善肝功能等方面明显优于对照组（维生素E），但是该研究没有对脂肪肝进行定量评价。目前国内没有应用吡格列酮对伴糖调节异常NAFLD患者的治疗研究。

小檗碱，又名黄连素，是中药黄连的主要成分,临床广泛用于消化道感染的治疗。2004年由中国医学科学院医药生物技术研究所所长蒋建东博士领导的课题组发现，小檗碱可通过转录后修饰机制稳定mRNA以增加LDL受体表达，从而发挥降低血脂作用[[[31]](#endnote-32)]，与目前使用的他汀类降血脂药物的作用机理完全不同，该研究发表在国际权威的[Nature Medicine杂志上](javascript:AL_get(this, 'jour', 'Nat Med.');)。由于脂肪肝与血脂代谢紊乱密切相关，小檗碱可能通过改善脂代谢从而对脂肪肝产生治疗作用。迄今国外尚未见到应用小檗碱治疗NAFLD的相关报道，国内有2个研究对小檗碱治疗NAFLD的疗效进行评价[[[32]](#endnote-33),[[33]](#endnote-34)]，结果显示小檗碱治疗后，NAFLD患者血糖、血脂、肝酶、BMI等指标较治疗前明显下降，患者耐受性好。但是这2个研究仅采用B超对肝脏脂肪含量进行评价，方法学不精确，样本量也比较小，未设立对照组。

我院在脂肪肝的防治过程中发现，经生活方式干预及吡格列酮治疗，NAFLD患者2－3个月左右即可出现明显的体重减轻及肝脏脂肪含量的下降（CT测定的肝脏脂肪含量治疗前为20%，治疗后接近0%）。故本研究采用**4个月作为脂肪肝治疗的疗程**。

1. **研究目的：**

选择伴有糖调节异常和新诊断2型糖尿病的非酒精性脂肪性肝病（NAFLD）患者，在生活方式干预的基础上，将受试者随机分为3组：单纯生活方式干预组；生活方式干预＋盐酸吡格列酮组（盐酸吡格列酮片15mg qd. po）；生活方式干预＋盐酸小檗碱组（盐酸小檗碱片0.5g tid. po），**进行为期16周的随机对照开放临床试验，**以质子磁共振波谱（^1^H MRS）方法测定治疗前后肝脏脂肪含量的变化，评价以上三种方法对受试者肝脏脂肪含量的变化和糖代谢改善的疗效及两种药物的安全性，分析肝脏脂肪含量的改变与血糖、血脂等代谢之间的关系，提出适合我国伴有糖代谢异常的NAFLD人群中降低肝脏脂肪含量的治疗方法。

**三、受试者条件**

**3.1 入选标准**

1. 已充分了解知情同意书，并签署知情同意书；
2. 年龄：≥18～≤65岁；
3. B超诊断为脂肪肝；
4. 空腹血糖≥5.6mmol/L和/或糖负荷后2小时血糖≥7.8mmol/L；
5. 糖尿病病程≤1年；
6. 近4周内未服过任何降糖药或接受胰岛素治疗；
7. 近4周内未服用调脂药者（他汀类，贝特类）；
8. 经改善生活方式和/或服用有效的降压药物，血压<160/100mmHg；
9. 曾服用益肝灵、熊脱氧胆酸、易善复、维生素E、复方玉芩胶囊、水飞蓟素等药物，已停药4周以上；
10. **^1^H MRS测定肝脏脂肪含量≥13%。**

**3.2 排除标准**

1) 有下列任何一项肝病史者：

慢性肝炎、肝硬化、肝癌、自身免疫性肝病、酒精性肝病、遗传性肝病等；

2） 肝功能显著异常：ALT或AST≥正常上限2倍；

3） HBsAg（＋）, 和/或HCV-Ab（＋）；

4) 1型糖尿病，特殊类型糖尿病及妊娠糖尿病患者,BMI<22kg/m^2^；

5) **糖尿病病程>1年；**

6） 曾接受或正在进行任何降糖药或胰岛素治疗的患者；

7） HbA1c>7.5% ；

8) 合并严重糖尿病并发症的患者（糖尿病酮症酸中毒、昏迷及有糖尿病昏迷征兆的患者；神经功能障碍、视网膜病变、肾功能障碍）；

9) 血清肌酐值在≥1.5mg/dL（133umol/L）的患者；

10）合并重症心脏疾病的患者（曾发生过心肌梗塞患者及心衰和/或严重心律失常）；

11）合并重症感染症、经历手术后6个月内、严重外伤患者；

12）饮酒（酒精量）：男性≥140g/周；女性≥70g/周；

13）24周内参加过其他临床试验的患者；

14）有同类药物过敏史的患者；

15）妊娠或可能妊娠及哺乳期或者，或试验期间希望怀孕的男性或女性患者；

16）因精神疾患不能依从该研究方案者；

17）不签署知情同意书者；

18）其他，研究者认为不适合作为试验对象者；

**19）血清甘油三酯≥5.0mmol/L者；**

**20）甲状腺疾病，包括甲状腺功能亢进症和甲状腺功能减退症。**

**3.3 剔除标准**

1. 妊娠；
2. 在随访期间患者收缩压≥180mmHg和或舒张压≥110mmHg；
3. 肝酶（ALT或AST）≥正常上限2倍；
4. 血肌酐≥1.5mg/dl(133umol/L) ；
5. 加服了干扰药物：包括：除吡格列酮外的所有降血糖药物、调脂药、具有降糖、降脂作用的中药等；

**四、干预方法与研究药物**

**4.1 单纯生活方式干预：**

4.1.1 限热卡饮食：筛选期记录连续3天饮食日记，计算每日平均热卡，然后根据平均值减去500千卡/天^[25]^；

4.1.2 运动指导：每周进行中等强度的有氧运动（心率达到最大心率的50－70％）至少150min，和/或每周较高强度的有氧运动（心率达到最大心率的70％）至少90分钟；每周至少运动3天，不运动天数不能超过连续2天[[[34]](#endnote-35)]。

最大心率计算公式：最大心率＝220－年龄。

**4.2 选择干预药物的依据**

4.2.1：盐酸吡格列酮（pioglitazone）片，商品名：艾汀（Actlns）

属于上市药物；

生产厂家：北京太洋药业有限公司；

规格：盐酸吡格列酮15 mg/片；

剂型：片剂；

选择依据：

- - - 1. 属于胰岛素增敏剂，临床已经用于治疗2型糖尿病，而参加本研究受试者均为存在糖代谢异常的NAFLD患者；
      2. 大规模PROactive研究旨在观察吡格列酮相对于安慰剂在2型糖尿病患者心脑血管事件二级预防中的作用，发现吡格列酮与安慰剂相比，可以减少心脑血管终点事件，同时发现，吡格列酮组患者ALT水平减少5％，而安慰剂组ALT水平升高8％[[[35]](#endnote-36)]，提示吡格列酮可以减少肝酶水平。
      3. 针对非酒精性脂肪性肝炎（NASH）的研究提示吡格列酮与安慰剂相比，可以明显减少NASH患者肝脏脂肪含量；改善NASH患者的生物学指标及肝脏组织学病变；降低NASH患者血糖水平及改善糖耐量，降低肝酶水平^[25]^。
      4. 2004年11月美国国家糖尿病、消化与肾脏病研究院（NIDDK）已经启动了吡格列酮 vs维生素E vs安慰剂治疗非糖尿病的非酒精性脂肪性肝炎Ⅲ期临床研究（PIVENS），为了缩小与国际研究水平的差距，我们需要尽快进行吡格列酮治疗NAFLD的临床试验研究。

4.2.2：盐酸小檗碱片（Berberine），商品名：盐酸小檗碱片

属于上市药物；

生产厂家：上海华氏制药有限公司；

规格：盐酸小檗碱片0.1g/片；

剂型：片剂；

选择依据：

1. 分子式：C_20_H_18_ClNO_4_·2H_2_O
2. 动物与人体研究提示小檗碱具有降低血糖、血脂作用；
3. 小檗碱可通过转录后修饰机制稳定mRNA以增加LDL受体表达，从而发挥降低血脂作用^[31]^，与目前使用的他汀类降血脂药物的作用机理完全不同，由于脂肪肝与血脂代谢紊乱密切相关，小檗碱可能通过改善脂代谢从而对脂肪肝产生治疗作用；
4. 国内有小样本研究显示小檗碱治疗后，NAFLD患者血糖、血脂、肝酶、BMI等指标较治疗前明显下降。

**五、治疗方法**

盐酸吡格列酮（pioglitazone）片，15mg qd. po.（1片/日, 早餐前30分钟口服）

盐酸小檗碱（Berberine）片，0.5g tid. po.（5片/次,3次/天，三餐前30分钟口服）

**疗程：治疗16周。**

**六、合并用药**

**禁止同时合并使用的药物**

在试验期间（观察期及治疗期），禁止合用以下药物：

1. 胰岛素制剂
2. 胰岛素增敏剂（罗格列酮Rosiglitazone）
3. 磺脲类药物（格列本脲Glibenclamide，格列吡嗪Glipizide，格列喹酮Gliquidone，

格列美脲Glimepiride，格列齐特Gliclazide等）

1. 双胍类药物（二甲双胍Metformin）
2. α-糖苷酶抑制剂（阿卡波糖Acarbose，伏格列波糖Voglibose）
3. 速效促胰岛素分泌药物（瑞格列奈Repaglinide，那格列奈Nateglinide）
4. 开发（含临床试验阶段）中的药物
5. 调脂药（他汀类、贝特类）
6. 针对脂肪肝的其他药物：益肝灵、熊脱氧胆酸、易善复、维生素E、复方玉芩胶囊、

水飞蓟素等药物。

**七、 试验药品的管理**

**7.1 试验药物的提供和储存**

该课题所用药物均为上市药物，方案中试验药物由我课题组用该课题科研经费向市场统一购买相同批号的药品。课题组专人负责药品储存和分发。试验过程中，试验药品由课题负责人指定专人负责，并保存在安全地方。

**7.2 试验药物的分发和管理**

满足所有入选标准，且与所有排除标准没有抵触的筛选合格的患者将在0周时，经过随机分组得到有药物编号的药盒。病人要在下次随访时退还未用的药物和包装盒。

主要研究者或研究者负责管理相应的受试者随机分配的试验用药，按照已定的方法正确用药。主要研究者或研究者需要调查试验药物的使用状况，并记录在病例报告表中。包括用药时间、用药量、有无中止、中断的状况，如果有中止：记录中止、中断的时间，中止、中断的理由。

**八、研究过程**

**8.1 流程图**

|  | **筛选期（V0）** | **V1** | **V2** | **V3** | **V4** | **V5** |
| --- | --- | --- | --- | --- | --- | --- |
|  | **-4周** | **0周** | **4周** | **8周** | **12周** | **16周** |
| 签署知情同意书 | ● |  |  |  |  |  |
| 初选表 （另附） | ● |  |  |  |  |  |
| 病史收集 | | | | | |  |
| 既往史与治疗史 | ● |  |  |  |  |  |
| 合并用药与伴随疾病 | ● | ● | ● | ● | ● | ● |
| 确定入选与排除标准 | ● | ● |  |  |  |  |
| 营养、运动量随访调查问卷 |  | ● |  |  |  | ● |
| 体检与化验 | | | | | |  |
| 身高 | ● |  |  |  |  |  |
| 体重、腰围、臀围  血压、心率、心律 | ● | ● | ● | ● | ● | ● |
| 空腹血糖+糖负荷后2h血糖 | ● |  | ● | ● | ● |  |
| OGTT（血糖＋胰岛素）、 |  | ● |  |  |  | ● |
| 血脂 | ● | ● |  |  |  | ● |
| 肝功能 | ● | ● | ● | ● | ● | ● |
| HbA1c | ● |  |  |  |  | ● |
| 血尿常规 |  | ● | ● | ● | ● | ● |
| 肾功能 | ● | ● | ● | ● | ● | ● |
| 尿妊娠试验 |  | ● |  |  |  | ● |
| 肝炎病毒标志物（HBsAb、HCV-Ab） | ● |  |  |  |  |  |
| 心电图 | ● |  |  |  |  | ● |
| 肝脏脂肪含量（MRS）±1周 | ● |  |  |  |  | ● |
| 肝脏脂肪含量 [B超（六院）] | ● |  |  |  |  | **●** |
| 随机分组 |  | ● |  |  |  |  |
| 健康教育、生活方式指导 |  | ● | ● | ● | ● | ● |
| 试验用药品发放 |  | ● | ● | ● | ● |  |
| 试验用药品回收及服药依从性评价 |  |  | ● | ● | ● | ● |
| 不良事件记录和评估 |  | ● | ● | ● | ● | ● |
| 预约下次随访 | ● | ● | ● | ● | ● | ● |

**8.2试验过程与观察项目**

**筛选期（V0）：**第－4周

签署知情同意书、完成初筛表、病史收集（既往史与治疗史、合并用药与伴随疾病、确定入选与排除标准）、人体学测量（身高、体重、腰围、臀围、血压、心率、心律）、化验[肝酶、肾功能、0h+服75g葡萄糖粉后2h血糖、HbA1c、肝炎病毒标志物（HBsAg、HCV-Ab）] 、心电图、肝脏脂肪含量[MRS＋改良B超（六院部分受试者）；

基线调查时已经进行药物治疗者需停药4周；

由营养师指导记录连续3天饮食日记。

预约下次随访。

**随访1（V1）**：第0周

记录合并用药与伴随疾病、填写营养、运动量随访调查问卷、人体学测量（体重、腰围、臀围、血压、心率、心律）、化验[肝功能、OGTT（血糖＋胰岛素）、血脂、血尿常规、肾功能、尿妊娠试验、其他可选检查]；

随机分组：入选后，根据随机表，所有患者被随机化分为三组：单纯生活方式干预组；生活方式干预＋盐酸吡格列酮组；生活方式干预＋盐酸小檗碱组，

试验用药品发放，**发药时嘱患者第2天早餐前开始服药，**共服用12周；

健康教育、生活方式指导（限制热卡饮食）：计算患者V0期间记录的连续三日平均摄入的热卡，减去500大卡/天作为每日摄入热卡的值。

获得化验结果后，**当天下午或晚上**由研究者判断患者是否仍然符合入选标准，符合者开始服药，不符合者退出本研究，电话通知患者回访并回收药物；

不良事件记录和评估；

预约下次随访。

**随访2（V2）：**第4周

记录合并用药与伴随疾病、人体学测量（体重、腰围、臀围、血压、心率、心律）、化验（肝酶、0h+2h血糖及胰岛素、血尿常规、肾功能）；

健康教育、生活方式指导（限制热卡饮食）；

试验用药品发放；

试验用药品回收及服药依从性评价；依从性应该>80%～<120%。

依从性计算公式：依从性＝（发药数量－回收药量）/应服药量×100％

不良事件记录和评估；

预约下次随访。

**随访3（V3）：**第8周

记录合并用药与伴随疾病、人体学测量（体重、腰围、臀围、血压、心率、心律）、化验（肝酶、0h+2h血糖及胰岛素、血尿常规、肾功能）；

健康教育、生活方式指导（限制热卡饮食）；

试验用药品发放；

试验用药品回收及服药依从性评价；依从性应该>80%～<120%。

依从性计算公式：依从性＝（发药数量－回收药量）/应服药量×100％

不良事件记录和评估；

预约下次随访。

**随访4（V4）：**第12周

记录合并用药与伴随疾病、人体学测量（体重、腰围、臀围、血压、心率、心律）、化验（肝酶、0h+2h血糖及胰岛素、血尿常规、肾功能）；

健康教育、生活方式指导（限制热卡饮食）；

试验用药品发放；

试验用药品回收及服药依从性评价；依从性应该>80%～<120%。

依从性计算公式：依从性＝（发药数量－回收药量）/应服药量×100％

不良事件记录和评估；

预约下次随访。

**随访5（V5）：**第16周 研究结束期

记录合并用药与伴随疾病、填写营养、运动量随访调查问卷、人体学测量（体重、腰围、臀围、血压、心率、心律）、化验[肝功能、OGTT（血糖＋胰岛素）、血脂、血尿常规、肾功能、尿妊娠试验、其他可选检查]；心电图、肝脏脂肪含量[MRS＋改良B超（部分受试者）]；

健康教育、生活方式指导；

试验用药品发放；

试验用药品回收及服药依从性评价；依从性应该>80%～<120%。

依从性计算公式：依从性＝（发药数量－回收药量）/应服药量×100％

不良事件记录和评估；

如需附加随访，预约随访日期。

**附加随访：**

若患者出现不良事件以及实验室检查值异常结果需要随访时，请记载有关事项（包括追踪项目、检查日期、以及检查结果）。

**8.3研究路线**

组1

单纯生活方式干预组（60例）

随机分组，发放相应药物

收集基线数据：

人体测量、代谢相关指标、

肝脏脂肪含量（MRS/B超）、安全相关指标

符合条件受试者180例

每4周随访一次，共16周

组2

生活方式干预+吡格列酮治疗组（60例）

组3

生活方式干预+小檗碱治疗组（60例）

收集与基线相同数据，统计分析

**九、病例数量与安排**

**9.1 样本量计算：**

根据主要研究终点（肝脏脂肪含量下降的程度）来计算样本量，该指标为计量数据，故样本计算采用如下公式：n1=n2=2[(t_α_+ t_2β_)s/δ]^2^

s：标准差，（国外研究数据提示NAFLD患者肝脏脂肪含量的标准差约为3－27％，我科前期研究数据提示NAFLD患者肝脏脂肪含量的标准差为6.87％）；

δ:研究者期望的实验组与对照组的差值，国外研究示吡格列酮治疗后NAFLD肝脏脂肪含量下降约10－25％^[25，26，27，28]^，我科前期研究数据提示NAFLD患者吡格列酮治疗后NAFLD肝脏脂肪含量下降约10-20％,本研究暂定为10％，假设生活方式干预组的脂肪含量下降为5％，则δ为5％。

β取单侧0.10，α取双侧0.05，因为是三组比较，实际α为双侧0.016。

计算得出每组样本数为： n=2[(2.41+ 1.282)×6.87%/5%]^2^=52例，共需156例。考虑15％的脱落病例，实际需入组180例病人。

**9.2病例安排：**

本研究与上海市交通大学附属第六人民医院内分泌科合作完成。

| 研究单位 | 生活方式干预组 | 生活方式干预＋吡格列酮组 | 生活方式干预＋小檗碱组 | 合计 |
| --- | --- | --- | --- | --- |
| 复旦大学附属  中山医院内分泌科 | 40例 | 40例 | 40例 | 120例 |
| 上海市交通大学附属  第六人民医院内分泌科 | 20例 | 20例 | 20例 | 60例 |

**十、临床观察项目和指标**

**10.1确定的检测指标**

1. 调查问卷：人口统计学指标、个人疾病史、疾病家族史、受教育程度、吸烟和饮酒、体力活动、膳食习惯和药物使用情况等；
2. 人体测量和体检：身高、体重、腰围和臀围，采用国际标准化方法测量血压，每次测3遍血压，心率及心律（脉搏触诊）；
3. 实验室测定（必做）：主要是：

(1)、空腹血糖(FPG)、负荷后2小时血糖（2hPG)、OGTT(5点血糖＋胰岛素)、血脂谱全套（TC、TG、HDL-c、LDL-c、ApoA、ApoB、ApoE、Lpa）；肝酶(ALT、AST、γ-GT、ALP) 、HbA1c、尿白蛋白/肌酐比值；

(2)、安全性相关实验室检查（血、尿常规检查；肝、肾功能；尿妊娠试验、ECG）

(3)、肝脏脂肪含量： ^1^H MRS、改良B超

**10.2观察终点**

主要终点：血糖水平（空腹血糖、糖负荷后2h血糖、OGTT葡萄糖及葡萄糖曲线下面积、HbA1c）；

血脂谱（TC、TG、HDL-c、LDL-c、ApoA、ApoB、ApoE、Lpa）；

肝酶(ALT、AST、γ-GT、ALP)。

次要终点：^1^H MRS测定的肝脏脂肪含量（％）变化；

胰岛素水平及曲线下面积变化。

**十一、非严重不良事件（NSAE）的观察**

**11.1 非严重不良事件的定义**

非严重不良事件是指使用药物过程中或用药后发生的，不可预见的医疗状况，或者使原有医疗状况恶化的一种事件，其发生与所用药物不一定有因果关系。不良医疗状况包括症状（如：恶心，胸痛），体征（如：心动过速，肝脏肿大）或异常检查结果（如：实验室检查，心电图检查）。

在临床研究中，NSAE可以包括任何时间所发生的任何不能预见的不适，包括筛选期或清洗期，甚至在还没有使用研究药物时。

**11.2 非严重不良事件的记录**

应当在整个研究过程中注意NSAE的收集。

以下是对每个NSAE应记录的变量：发作和停止时间、轻重程度、采取的措施、与药物使用的因果关系（有或无）、结果以及是否是严重不良事件。

**11.3 非严重不良事件的严重程度分级：**

1＝轻度（有症状或体征，但能忍受）

2＝中度（感到不适且影响正常活动）

3＝重度（无力进行正常活动）

严重度和严重不良事件的区分很重要。严重度是对不良事件程度的测定，而严重不良事件需根据下述12.1标准定义。比如：持续数小时的恶心，其程度可能是严重的，但不是严重不良事件。另一方面仅导致局限性功能丧失的中风可以被认为是轻度中风，但应认为是严重不良事件。

试验结束时未解决的NSAE应继续随访，其随访间隔与延续时间视医疗需要而定。对试验结束时或受试者退出研究时未解决的不良事件，应随访至NSAE恢复正常，或至研究者认为不需要再继续随访时。

一旦发生怀孕，必须按程序报告。怀孕本身并不是一个不良事件，除非怀疑研究药物可能与口服避孕药相互作用而影响后者的效应。

**11.4 非严重不良事件与研究药物关系的判断标准**

参照以下6级分类标准评定。其中第1－3级为不良反应。

1. 肯定：用药及反应发生时间顺序合理；停药反应停止，或迅速减轻或好转（根据机体免疫状态某些ADR反应可出现在停药数天以后）；再次使用，反应再现，并可能明显加重（即激发试验阳性）；同时有文献资料佐证，并已除外原患疾病等其它混杂因素影响。
2. 很可能：无重复用药史，余同“1、肯定”，或虽然有合并用药，但基本可排除合并用药导致反应发生的可能性。
3. 可能：用药与反应发生时间关系密切，同时有文献资料佐证；但引发NSAE的药品不止一种，或原患疾病病情进展因素不能除外。
4. 可能无关：NSAE与用药时间相关性不密切，反应表现与已知该药物的不良反应不相吻合，原患疾病发展同样可能有类似的临床表现。
5. 无关：反应出现不符合用药后合理的时间顺序，反应有符合非试验药物已知的反应类型；受试者的临床状态或其它的治疗方式可能产生该反应，疾病状态改善或停止其它治疗方式反应消除，重复使用其它治疗方法反应出现。
6. 无法评价：报表内容填写不齐全，等待补充后再评价，或因果关系难以定论，缺乏文献资料佐证；报表缺项太多因果关系难以定论，资料又无法补充。

**11.5 本试验NSAE的处理**

如有发生，应判断其与服药有关、可能有关或无关，并统计发生率。不良反应轻者可继续用药并密切观察，中等者药量可减轻，严重者应停药并及时采取相应治疗，并向课题研究负责人报告。对不良反应的严重程度、持续时间、处理方法和转归应加以说明。试验期间如同时用其它药物应记录。

**十二、严重不良事件（SAE）**

**12.1严重不良事件的判定**

严重不良事件是指在研究阶段（如：筛选法，治疗期，清洗期和随访期）使用任何剂量的研究药物，对照药或安慰剂导致的符合以下的一项或多项的标准的不良事件：

1. 导致死亡的；
2. 即刻危及生命的；
3. 需要住院或住院时间延长的；
4. 导致持续性或明显的伤残/机能丧失；
5. 先天畸形/缺陷；
6. 重大的医疗事件，可能使受试者受到损伤或需要采取医疗干预来防止上述所列情况

发生。

**12.2 严重不良事件的处理**

在研究过程中发现任何严重不良事件，研究相关人员都必须在24h内通知课题小组及负责人和主要研究人员。

如由非严重不良事件进展为严重不良事件，该事件及相关追踪报告必须在发现24h内报告给课题负责人。

所有的严重不良事件无论是否与研究药物相关都应报告，并应记录在病例报告表的不良事件报告表中，记录严重不良反应的发生时间、严重程度、持续时间、采取的措施等。根据中国相关法规的要求，研究者有责任通知伦理委员会或/和政府法规事务部门。

在报告相关人员的同时，根据情况及时采取相应的治疗措施。

联系方式如表：

| 单位 | 联系人 | 电话 |
| --- | --- | --- |
| 复旦大学附属中山医院内分泌科 | 颜红梅 | 13788961990－612428 |
| 复旦大学附属中山医院内分泌科 | 刘蒙 | 13788961990－670692 |
| 复旦大学附属中山医院内分泌科 | 高鑫 | 13788961990－680362 |
| 上海交通大学附属第六人民医院内分泌科 | 贾伟平 | 签署二级合同后确定联系人 |

**十三、随机化方法**

本试验为随机、开放、对照的临床试验。采用分中心区组随机化方法，随机表由复旦大学公共卫生学院统计教研室提供，（利用SAS8 软件生成），由与本次临床试验无关人员完成随机化工作。根据随机安排表，每个病人制作一个标有治疗编号的随机信封。病人入选后，按入选顺序分配治疗编号，并根据相应随机信封的安排给予处理。发药结束后，随机分配表一式两份由研究主要负责人复旦大学附属中山医院内分泌科高鑫教授和研究小组保存。

**十四、数据管理**

1. **病例报告表的填写与移交**

病例报告表由研究者填写，每个入选病例必须完成病例报告表。完成的病例报告表由临床监察员审查后，第一联移交数据管理员，进行数据录入与管理工作。

1. **数据的录入与修改**

复旦大学公共卫生学院统计教研室负责有关计算机设备，软件、资料输录员培训考核。统一采用软件EPIDATA3.2进行数据管理，采取双份独立录入并校对，直至两份数据库内容完全一致。

对病例报告表中存在的疑问，数据管理员将产生疑问解答表(DRQ)，并通过临床监察员向研究者发出询问，研究者应尽快解答并返回，数据管理员根据研究者的回答进行数据修改、确认与录入，必要时可以再次发出DRQ。

1. **数据锁定**

所有数据录入完成，疑问表均已解决、数据清理干净后，由课题负责人、主要研究者、数据管理员、统计分析人员对数据进行审核，确定各个分析人群，在审核完成并确认建立的数据库正确后，对数据进行锁定。锁定后的数据文件不再做改动。数据锁定之后发现的问题，经确认后在统计分析程序中进行修正。

1. **数据处理**

在研究数据全部录入并锁定后，将数据库交统计分析人员按统计计划书要求进行统计分析。完成统计分析后，由统计分析人员写出统计分析报告，交本试验的主要研究者写出研究报告。

**十五、统计分析**

**15.1 分析人群和分析工具**

将有三个人群参与分析：全分析集、符合方案集和安全集。

对主要疗效指标将同时采用全分析集和符合方案集分析；所有次要疗效指标，将使用全分析集分析；安全性评价将采用安全集分析。基线的均衡性评估也将采用全分析集。

- **全分析集**(FAS，Full Analysis Set)：指尽可能接近符合意向性治疗原则(ITT原则)的理想的受试者集。该数据集是从所有随机化的受试者中，以最少的和合理的方法剔除受试者后得出的。它包括经过随机化，有有效的基线值，至少服用过一次研究药物并且有用药后疗效记录的所有受试者。
- **符合方案分析集**（PPS，Per Protocol Set）：它是全分析集的更加符合方案的子集，这些受试者对方案更具有依从性，依从性包括：按方案要求完成全部研究的随访，主要指标可以测定，用药的依从性在80%—120%，没有对试验方案的重大违背。
- **安全集**(SS，Safty Set)：安全性数据集包括所有经过随机化，并至少接受一次药物治疗的受试者，并且有用药后的安全性评价资料。

在用全分析集分析时，对主要指标的缺失值，不进行观察值结转。

各个分析集将在数据库所锁定以前由主要研究者、数据管理员和统计师以及申办者共同讨论决定。

统计分析采用SAS8.02软件进行。

**15.2疗效评价：**

疗效评价的主要终点包括

- - 血糖水平（空腹血糖、糖负荷后2h血糖、OGTT葡萄糖及葡萄糖曲线下面积、HbA1c）
  - 血脂谱（TC、TG、HDL-c、LDL-c、ApoA、ApoB、ApoE、Lpa）
  - 肝酶(ALT、AST、γ-GT、ALP)

次要终点包括

- - ^1^H MRS测定的肝脏脂肪含量（％）变化；
  - 胰岛素水平及曲线下面积变化；
  - 因无效退出的比例

统计分析包括统计描述和统计推断。定量指标的统计描述采用均数、标准差、中位数、最大最小值等指标；定性指标通过频数表、构成比进行描述。统计推断包括区间估计和假设检验。

假设检验包括组内比较和组间比较。定量资料的组内比较为各个研究组治疗前后的比较，采用配对t检验或配对符号秩检验；组间比较包括各次随访时三组间（单纯生活方式干预、生活方式干预加吡格列酮或生活方式干预加小檗碱）的比较和三组治疗前后改变量之间的比较。采用方差分析的方法进行检验，如果p<0.05,可以认为三种治疗方式的总体均数不全相同，进而采用方差分析的两两比较方法进一步分析。如果资料非正态，可采用秩变换方法进行分析。

定性资料的比较将采用卡方检验。如为有序分类资料，将采用CMH卡方进行假设检验。

对主要疗效指标（肝脏脂肪含量％变化），采用可信区间进行优效性检验，因有两个试验组（生活方式干预加吡格列酮或生活方式干预加小檗碱），需进行两次比较，故对α进行调整，取α/2即0.0125计算单侧可信区间。各个用药组的可信区间下限如高于对照组的可信区间下限，按照α＝0.025的检验水准可认为药物组效果优于对照组。

**15.3安全性评价**

安全性评价内容包括：

- 一般不良事件（NSAE）
- 严重不良事件(SAE)
- 实验室检查指标
- 心电图

计算并比较每组非严重不良事件的发生率，与研究药物有关(肯定有关、很可能有关、可能有关)的NSAE发生率。NASE发生率的比较采用卡方检验。

NSAE将分组描述和列表，与用药有关的NSAE也要列出。

列表总结并比较两组NSAE的强度、与药物的关系、采取的措施，因NSAE退出的比例。

对于严重不良事件主要列表总结各组SAE发生的情况，包括SAE名称、持续时间、后果、对主要研究药物采取的措施、与药物的关系等。

各实验室检查指标将根据各个实验室提供的参考值范围确定为正常(含异常但无临床意义)或异常(指有临床意义的异常)。列表总结发生异常变化(指研究开始时正常、研究中或结束时异常)的实验室指标。列表总结发生异常变化的心电图。

**15.4 其他分析**

包括病例完成情况描述、基线均衡性分析，用药的依从性描述、合并用药分析。

脱落分析：列表描述各组总脱落率、脱落原因和由于不良事件而导致的脱落，采用卡方检验进行比较。

基础值的均衡性分析：采用方差分析或卡方检验来比较人口学资料和其他基础值指标，以衡量两组均衡性。

用药的依从性：描述并比较（采用方差分析或Wilcoxon秩和检验）各组的依从性，并分组描述依从性差的原因，采用卡方检验或Fisher确切概率法。

合并用药：描述研究中发生的违禁用药情况。

**十六、伦理要求**

1. 遵循赫尔辛基宣言(1996年版)；
2. 每例受试者均在知情同意书上签字；
3. 全部试验过程受复旦大学附属中山医院伦理委员会的监督。

**十七、资料保存**

为保证复旦大学附属中山医院内分泌科的评价与监督，各中心的研究者应保存所有的研究资料（包括原始记录、所有原始的有签名的患者知情同意书、所有CRF、药物分发的详细记录等）。保存期10年。本次临床研究的所有资料，所有权属于复旦大学附属中山医院组织的课题协作组。

**十八、总结**

本次临床研究的数据管理和统计分析将由复旦大学附属中山医院内分泌科负责，并委托复旦大学公共卫生学院统计教研室完成，另有补充协议。各中心资料由复旦大学附属中山医院内分泌科汇总统一分析，完成临床研究统计总结报告。

**十九、时间安排**

| 时间 | 内 容 |
| --- | --- |
| 2007年12月 | 伦理审批；美国NIH注册 |
| 2008年1月 | 临床研究启动；工作人员培训；表格与资料的准备；  药物、试管等物品的准备 |
| 2008年2月 | 第一例入组 |
| 2008年5月 | 完成第一例随访 |
| 2008年8月 | 最后一例入组 |
| 2008年11月30日 | 完成最后一例随访 |
| 2008年12月－2009年5月 | 资料整理，统计分析 |

**二十、其他事宜**

协作单位在临床试验期间接受牵头单位检查人员的定期探访。临床试验资料等经复旦大学附属中山医院内分泌科评审和同意后，方可发表学术论文。

**二十一、组织领导结构**

1. 组织领导：

课题主要负责人：高鑫；

课题组成员：颜红梅、刘蒙、桂明辉、常薪霞、俞璐、吴丹、花霞、曹萍、攸然

1. 随访调查质量控制负责人（监察员）：颜红梅
2. 统计分析人员：复旦大学公共卫生学院统计教研室 金丕焕、邓伟
3. 实验室质量控制组：潘柏申
4. 所有调查人员要诚实守信，讲求信誉。

**参考文献**

1. Centro Studi Fegato, AREA Science Park, Basovizza etal. Prevalence of and risk factors for nonalcoholic fatty liver disease: the Dionysos nutrition and liver study. Hepatology. 2005 Jul;42(1):44-52. [↑](#endnote-ref-2)
2. Leon A. Adams, Paul Angulo, Keith D et al. Nonalcoholic fatty liver disease. CMAJ. 2005; 172(7): 899 -905. [↑](#endnote-ref-3)
3. Jian-Gao Fan1, Jun Zhu1, Xing-Jian Li etal. Prevalence of and risk factors for fatty liver in a general population of Shanghai, China. Journal of Hepatology.2005.43:508–514. [↑](#endnote-ref-4)
4. Bugianesi E, Leone N, Vanni E, et al. Expanding the natural history of nonalcoholic steatohepatitis: from cryptogenic cirrhosis to hepatocellular carcinoma. Gastroenterology.2002,123: 134-40. [↑](#endnote-ref-5)
5. Nannipieri M, Haffner SM, , Ferrannini E, et al. Liver enzymes, the metabolic syndrome and incident diabetes: The mexico city Diabetes Study. Diabetes Care, 2005,28:1757-1762. [↑](#endnote-ref-6)
6. Marchesini G, Bugianesi E, Forlani G, et al. Nonalcoholic fatty liver, steatohepatitis, and the metabolic syndrome. Hepatology.2003;37: 917-23. [↑](#endnote-ref-7)
7. Sargin, Mehmet; Uygur–Bayramiçli, Oya; Sargin, Halu, et al. Association of Nonalcoholic Fatty Liver Disease With Insulin Resistance: Is OGTT Indicated in Nonalcoholic Fatty Liver Disease? [J Clin Gastroenterol.](javascript:AL_get(this, 'jour', 'J Clin Gastroenterol.');) 2003;37(5):399-402. [↑](#endnote-ref-8)
8. 颜红梅，高鑫，刘蒙等.NAFLD与代谢综合征关系. 中华糖尿病杂志,2006;14:326-328 [↑](#endnote-ref-9)
9. Vozarova B, Stefan N, Lindsay RS, et al. High alanine aminotransferase is associated with decreased hepatic insulin sensitivity and predicts the development of type 2 diabetes. Diabetes.2002,51: 1889 -1895 [↑](#endnote-ref-10)
10. Hanley AJ, Williams K, Festa A, et al. Insulin resistance atherosclerosis study. Elevations in markers of liver injury and risk of type 2 diabetes: the insulin resistance atherosclerosis study. Diabetes. 2004,53:2623-2632. [↑](#endnote-ref-11)
11. Perry IJ, Wannamethee SG, Shaper AG. Prospective study of serum gamma-glutamyltransferase and risk of NIDDM. Diabetes Care. 1998,21:732-737. [↑](#endnote-ref-12)
12. Lee DH, Ha MH, Kim JH, et al. Gamma-glutamyltransferase and diabetes–a 4 year follow-up study. Diabetologia. 2003,46:359-364. [↑](#endnote-ref-13)
13. Lee DH, Jacobs DR Jr, Gross M, et al. Gamma-glutamyltransferase is a predictor of incident diabetes and hypertension: the Coronary Artery Risk Development in Young Adults (CARDIA) Study. Clin Chem. 2003,49:1358-1366. [↑](#endnote-ref-14)
14. Lee DH, Silventoinen K, Jacobs DR Jr, et al. Gamma-Glutamyltransferase, obesity, and the risk of type 2 diabetes: observational cohort study among 20,158 middle-aged men and women. .J Clin Endocrinol Metab. 2004,89: 5410-5414.. [↑](#endnote-ref-15)
15. Sattar N, McConnachie A, Ford I, et al. Serial metabolic measurements and conversion to type 2 diabetes in the west of Scotland coronary prevention study: specific elevations in alanine aminotransferase and triglycerides suggest hepatic fat accumulation as a potential contributing factor. Diabetes.2007,56: 984-991. [↑](#endnote-ref-16)
16. Schindhelm RK, Dekker JM, Nijpels G, et al. Alanine aminotransferase predicts coronary heart disease events: A 10-year follow-up of the Hoorn Study. Atherosclerosis.2007,191:391-396. [↑](#endnote-ref-17)
17. Matteoni CA, Younossi ZM, Gramlich T, et al. Non-alcoholic fatty liver disease: a spectrum of clinical and pathological severity. Gastroenterology.1999,116:1413-1419. [↑](#endnote-ref-18)
18. Adams LA, Lymp JF, St Sauver J, et al. The natural history of non-alcoholic fatty liver disease: a population-based cohort study. Gastroenterology. 2005;129:113–121. [↑](#endnote-ref-19)
19. Carsten Thomsen, Ulrik Becker, Kjeld Winkler et al. Quantification of liver fat using magnetic resonance spectroscopy. [Magnetic Resonance Imaging](http://www.sciencedirect.com/science?_ob=JournalURL&_cdi=5112&_auth=y&_acct=C000053195&_version=1&_urlVersion=0&_userid=1508387&md5=56fcbc02832a203594bbd6aa80fd9ea5). 1994;12(3):487-495 [↑](#endnote-ref-20)
20. 刘蒙，高鑫, 饶圣祥等. 质子磁共振波谱分析（^1^H MRS）精确测定肝脏脂肪含量方法建立与初步研究.待发表 [↑](#endnote-ref-21)
21. [Lirussi F](http://www.ncbi.nlm.nih.gov/sites/entrez?Db=pubmed&Cmd=Search&Term="Lirussi F"%5BAuthor%5D&itool=EntrezSystem2.PEntrez.Pubmed.Pubmed_ResultsPanel.Pubmed_RVAbstract), [Azzalini L](http://www.ncbi.nlm.nih.gov/sites/entrez?Db=pubmed&Cmd=Search&Term="Azzalini L"%5BAuthor%5D&itool=EntrezSystem2.PEntrez.Pubmed.Pubmed_ResultsPanel.Pubmed_RVAbstract), [Orando S](http://www.ncbi.nlm.nih.gov/sites/entrez?Db=pubmed&Cmd=Search&Term="Orando S"%5BAuthor%5D&itool=EntrezSystem2.PEntrez.Pubmed.Pubmed_ResultsPanel.Pubmed_RVAbstract),et al. Antioxidant supplements for non-alcoholic fatty liver disease and/or steatohepatitis. [Cochrane Database Syst Rev.](javascript:AL_get(this, 'jour', 'Cochrane Database Syst Rev.');) 2007,24;(1):CD004996. [↑](#endnote-ref-22)
22. [Angelico F](http://www.ncbi.nlm.nih.gov/sites/entrez?Db=pubmed&Cmd=Search&Term="Angelico F"%5BAuthor%5D&itool=EntrezSystem2.PEntrez.Pubmed.Pubmed_ResultsPanel.Pubmed_RVAbstract), [Burattin M](http://www.ncbi.nlm.nih.gov/sites/entrez?Db=pubmed&Cmd=Search&Term="Burattin M"%5BAuthor%5D&itool=EntrezSystem2.PEntrez.Pubmed.Pubmed_ResultsPanel.Pubmed_RVAbstract), [Alessandri C](http://www.ncbi.nlm.nih.gov/sites/entrez?Db=pubmed&Cmd=Search&Term="Alessandri C"%5BAuthor%5D&itool=EntrezSystem2.PEntrez.Pubmed.Pubmed_ResultsPanel.Pubmed_RVAbstract),et al. Drugs improving insulin resistance for non-alcoholic fatty liver disease and/or non-alcoholic steatohepatitis. [Cochrane Database Syst Rev.](javascript:AL_get(this, 'jour', 'Cochrane Database Syst Rev.');) 2007,24;(1):CD005166 [↑](#endnote-ref-23)
23. [Lirussi F](http://www.ncbi.nlm.nih.gov/sites/entrez?Db=pubmed&Cmd=Search&Term="Lirussi F"%5BAuthor%5D&itool=EntrezSystem2.PEntrez.Pubmed.Pubmed_ResultsPanel.Pubmed_RVAbstract), [Mastropasqua E](http://www.ncbi.nlm.nih.gov/sites/entrez?Db=pubmed&Cmd=Search&Term="Mastropasqua E"%5BAuthor%5D&itool=EntrezSystem2.PEntrez.Pubmed.Pubmed_ResultsPanel.Pubmed_RVAbstract), [Orando S](http://www.ncbi.nlm.nih.gov/sites/entrez?Db=pubmed&Cmd=Search&Term="Orando S"%5BAuthor%5D&itool=EntrezSystem2.PEntrez.Pubmed.Pubmed_ResultsPanel.Pubmed_RVAbstract),et al. Probiotics for non-alcoholic fatty liver disease and/or steatohepatitis. [Cochrane Database Syst Rev.](javascript:AL_get(this, 'jour', 'Cochrane Database Syst Rev.');) 2007,24;(1):CD005165. [↑](#endnote-ref-24)
24. [Orlando R](http://www.ncbi.nlm.nih.gov/sites/entrez?Db=pubmed&Cmd=Search&Term="Orlando R"%5BAuthor%5D&itool=EntrezSystem2.PEntrez.Pubmed.Pubmed_ResultsPanel.Pubmed_RVAbstract), [Azzalini L](http://www.ncbi.nlm.nih.gov/sites/entrez?Db=pubmed&Cmd=Search&Term="Azzalini L"%5BAuthor%5D&itool=EntrezSystem2.PEntrez.Pubmed.Pubmed_ResultsPanel.Pubmed_RVAbstract), [Orando S](http://www.ncbi.nlm.nih.gov/sites/entrez?Db=pubmed&Cmd=Search&Term="Orando S"%5BAuthor%5D&itool=EntrezSystem2.PEntrez.Pubmed.Pubmed_ResultsPanel.Pubmed_RVAbstract),et al. Bile acids for non-alcoholic fatty liver disease and/or steatohepatitis. [Cochrane Database Syst Rev.](javascript:AL_get(this, 'jour', 'Cochrane Database Syst Rev.');) 2007,24;(1):CD005160 [↑](#endnote-ref-25)
25. [Belfort R](../../../../K:%5C开题%5C脂肪肝短期干预研究%5Cpioglitazone%20and%20fatty%20liver%5C%3FDb=pubmed&Cmd=Search&Term=%22Belfort%20R%22%5BAuthor%5D&itool=EntrezSystem2.PEntrez.Pubmed.Pubmed_ResultsPanel.Pubmed_RVAbstract), [Harrison SA](../../../../K:%5C开题%5C脂肪肝短期干预研究%5Cpioglitazone%20and%20fatty%20liver%5C%3FDb=pubmed&Cmd=Search&Term=%22Harrison%20SA%22%5BAuthor%5D&itool=EntrezSystem2.PEntrez.Pubmed.Pubmed_ResultsPanel.Pubmed_RVAbstract), [Brown K](../../../../K:%5C开题%5C脂肪肝短期干预研究%5Cpioglitazone%20and%20fatty%20liver%5C%3FDb=pubmed&Cmd=Search&Term=%22Brown%20K%22%5BAuthor%5D&itool=EntrezSystem2.PEntrez.Pubmed.Pubmed_ResultsPanel.Pubmed_RVAbstract),et al. A placebo-controlled trial of pioglitazone in subjects with nonalcoholic steatohepatitis. [N Engl J Med.](javascript:AL_get(this, 'jour', 'N Engl J Med.');) 2006;355(22):2297-307. [↑](#endnote-ref-26)
26. [Lutchman G](../../../../K:%5C开题%5C脂肪肝短期干预研究%5Cpioglitazone%20and%20fatty%20liver%5C%3FDb=pubmed&Cmd=Search&Term=%22Lutchman%20G%22%5BAuthor%5D&itool=EntrezSystem2.PEntrez.Pubmed.Pubmed_ResultsPanel.Pubmed_RVAbstract), [Promrat K](../../../../K:%5C开题%5C脂肪肝短期干预研究%5Cpioglitazone%20and%20fatty%20liver%5C%3FDb=pubmed&Cmd=Search&Term=%22Promrat%20K%22%5BAuthor%5D&itool=EntrezSystem2.PEntrez.Pubmed.Pubmed_ResultsPanel.Pubmed_RVAbstract), [Kleiner DE](../../../../K:%5C开题%5C脂肪肝短期干预研究%5Cpioglitazone%20and%20fatty%20liver%5C%3FDb=pubmed&Cmd=Search&Term=%22Kleiner%20DE%22%5BAuthor%5D&itool=EntrezSystem2.PEntrez.Pubmed.Pubmed_ResultsPanel.Pubmed_RVAbstract),et al. Changes in serum adipokine levels during pioglitazone treatment for nonalcoholic steatohepatitis: relationship to histological improvement. [Clin Gastroenterol Hepatol.](javascript:AL_get(this, 'jour', 'Clin Gastroenterol Hepatol.');) 2006;4(8):1048-52. [↑](#endnote-ref-27)
27. [Sanyal AJ](../../../../K:%5C开题%5C脂肪肝短期干预研究%5Cpioglitazone%20and%20fatty%20liver%5C%3FDb=pubmed&Cmd=Search&Term=%22Sanyal%20AJ%22%5BAuthor%5D&itool=EntrezSystem2.PEntrez.Pubmed.Pubmed_ResultsPanel.Pubmed_RVAbstract), [Mofrad PS](../../../../K:%5C开题%5C脂肪肝短期干预研究%5Cpioglitazone%20and%20fatty%20liver%5C%3FDb=pubmed&Cmd=Search&Term=%22Mofrad%20PS%22%5BAuthor%5D&itool=EntrezSystem2.PEntrez.Pubmed.Pubmed_ResultsPanel.Pubmed_RVAbstract), [Contos MJ](../../../../K:%5C开题%5C脂肪肝短期干预研究%5Cpioglitazone%20and%20fatty%20liver%5C%3FDb=pubmed&Cmd=Search&Term=%22Contos%20MJ%22%5BAuthor%5D&itool=EntrezSystem2.PEntrez.Pubmed.Pubmed_ResultsPanel.Pubmed_RVAbstract),et al. A pilot study of vitamin E versus vitamin E and pioglitazone for the treatment of nonalcoholic steatohepatitis. [Clin Gastroenterol Hepatol.](javascript:AL_get(this, 'jour', 'Clin Gastroenterol Hepatol.');) 2004;2(12):1107-15. [↑](#endnote-ref-28)
28. [Promrat K](../../../../K:%5C开题%5C脂肪肝短期干预研究%5Cpioglitazone%20and%20fatty%20liver%5C%3FDb=pubmed&Cmd=Search&Term=%22Promrat%20K%22%5BAuthor%5D&itool=EntrezSystem2.PEntrez.Pubmed.Pubmed_ResultsPanel.Pubmed_RVAbstract), [Lutchman G](../../../../K:%5C开题%5C脂肪肝短期干预研究%5Cpioglitazone%20and%20fatty%20liver%5C%3FDb=pubmed&Cmd=Search&Term=%22Lutchman%20G%22%5BAuthor%5D&itool=EntrezSystem2.PEntrez.Pubmed.Pubmed_ResultsPanel.Pubmed_RVAbstract), [Uwaifo GI](../../../../K:%5C开题%5C脂肪肝短期干预研究%5Cpioglitazone%20and%20fatty%20liver%5C%3FDb=pubmed&Cmd=Search&Term=%22Uwaifo%20GI%22%5BAuthor%5D&itool=EntrezSystem2.PEntrez.Pubmed.Pubmed_ResultsPanel.Pubmed_RVAbstract),et al. A pilot study of pioglitazone treatment for nonalcoholic steatohepatitis. [Hepatology.](javascript:AL_get(this, 'jour', 'Hepatology.');) 2004;39(1):188-96. [↑](#endnote-ref-29)
29. [Yoneda M](http://www.ncbi.nlm.nih.gov/sites/entrez?Db=pubmed&Cmd=Search&Term="Yoneda M"%5BAuthor%5D&itool=EntrezSystem2.PEntrez.Pubmed.Pubmed_ResultsPanel.Pubmed_RVAbstract), [Endo H](http://www.ncbi.nlm.nih.gov/sites/entrez?Db=pubmed&Cmd=Search&Term="Endo H"%5BAuthor%5D&itool=EntrezSystem2.PEntrez.Pubmed.Pubmed_ResultsPanel.Pubmed_RVAbstract), [Nozaki Y](http://www.ncbi.nlm.nih.gov/sites/entrez?Db=pubmed&Cmd=Search&Term="Nozaki Y"%5BAuthor%5D&itool=EntrezSystem2.PEntrez.Pubmed.Pubmed_ResultsPanel.Pubmed_RVAbstract), et al.Life Style-Related Diseases of the Digestive System: Gene Expression in Nonalcoholic Steatohepatitis Patients and Treatment Strategies.[J Pharmacol Sci.](javascript:AL_get(this, 'jour', 'J Pharmacol Sci.');) 2007,105: 151-156. [↑](#endnote-ref-30)
30. 张云良, 张润兰, 王君等. 吡格列酮治疗2型糖尿病合并脂肪肝疗效观察. 中国医师杂志，2007，9（5）:580-582. [↑](#endnote-ref-31)
31. [Kong W](http://www.ncbi.nlm.nih.gov/sites/entrez?Db=pubmed&Cmd=Search&Term="Kong W"%5BAuthor%5D&itool=EntrezSystem2.PEntrez.Pubmed.Pubmed_ResultsPanel.Pubmed_RVAbstractPlus), [Wei J](http://www.ncbi.nlm.nih.gov/sites/entrez?Db=pubmed&Cmd=Search&Term="Wei J"%5BAuthor%5D&itool=EntrezSystem2.PEntrez.Pubmed.Pubmed_ResultsPanel.Pubmed_RVAbstractPlus), [Abidi P](http://www.ncbi.nlm.nih.gov/sites/entrez?Db=pubmed&Cmd=Search&Term="Abidi P"%5BAuthor%5D&itool=EntrezSystem2.PEntrez.Pubmed.Pubmed_ResultsPanel.Pubmed_RVAbstractPlus), et al. Berberine is a novel cholesterol-lowering drug working through a unique mechanism distinct from statins.[Nat Med.](javascript:AL_get(this, 'jour', 'Nat Med.');) 2004,10(12):1344-51. [↑](#endnote-ref-32)
32. 魏敬, 吴锦丹, 蒋建东等. 盐酸小檗碱治疗2型糖尿病合并脂肪肝的临床研究.中西医结合肝病杂志,2004,14(6):334-336. [↑](#endnote-ref-33)
33. 刘才乐,张少君,罗禅清等.盐酸小聚碱治疗非酒精性脂肪肝34例.实用医学杂志, 2006, 22(21): 2519 -2520. [↑](#endnote-ref-34)
34. American Diabetes Association. Standards of Medical Care in Diabetes—2007. Diabetes Care 2007 30: S4-41. [↑](#endnote-ref-35)
35. John A Dormandy, Bernard Charbonnel, David J A Eckland,et al. Secondary prevention of acrovascular events in patients with type 2 diabetes in the PROactive Study (PROspective pioglitAzone Clinical Trial In macroVascular Events): a randomised controlled trial. The Lancet. 2005,366: 1279-1289. [↑](#endnote-ref-36)
