# Supplementary material for: Efficacy of Berberine in Patients with Non-Alcoholic Fatty Liver Disease
Source: PLoS One. 2015 Aug 7;10(8):e0134172. doi: 10.1371/journal.pone.0134172 (PMC4529214; doi:10.1371/journal.pone.0134172)
Supplement: S1 Table — (DOCX) [file pone.0134172.s006.docx]

| Supplement 3 Compliance with lifestyle intervention and medication | | | |  |
| --- | --- | --- | --- | --- |
|  | LSI group | LSI plus PGZ group | LSI plus BBR group | P value |
| Lifestyle compliance,n(%) | 49(92.5%) | 45(95.8%) | 52(94.5%) | 0.905 |
| Medication compliance,n(%) | - | 44(93.6%) | 51(92.7%) | 0.881 |
