# Supplementary material for: Efficacy of Berberine in Patients with Non-Alcoholic Fatty Liver Disease
Source: PLoS One. 2015 Aug 7;10(8):e0134172. doi: 10.1371/journal.pone.0134172 (PMC4529214; doi:10.1371/journal.pone.0134172)
Supplement: S2 Table — (DOCX) [file pone.0134172.s007.docx]

| Supplement 4 Drug-related Adverse Events | |  |  |
| --- | --- | --- | --- |
| Adverse events | LSI group | LSI plus PGZ group | LSI plus BBR group |
| Body as a whole |  |  |  |
| Headache | 0 | 1(5.26%) | 0 |
| Pain (unspecified) | 0 | 6(31.58%) | 0 |
| Discomfort | 0 | 1(5.26%) | 1(2.38%) |
| Fatigue | 0 | 3(15.79%) | 1(2.38%) |
| Itching | 0 | 0 | 1(2.38%) |
| Digestive system |  |  |  |
| Dyspepsia | 0 | 0 | 13(30.95%) |
| Nausea | 0 | 1(5.26%) | 4(9.52%) |
| Diarrhea | 0 | 1(5.26%) | 11(26.19%) |
| Abdominal pain | 0 | 0 | 1(2.38%) |
| hunger | 0 | 0 | 2(4.76%) |
| Constipation | 0 | 0 | 6(14.29%) |
| Circulatory system |  |  |  |
| Chest tightness | 0 | 1(5.26%) | 0 |
| Palpitation | 0 | 2(10.53%) | 0 |
| Hematological system |  |  |  |
| Leukopenia | 0 | 1(5.26%) | 1(2.38%) |
| Urogenital system |  |  |  |
| Proteinuria | 0 | 0 | 1(2.38%) |
| Menstruation | 0 | 1(5.26%) | 0 |
| Endocrine system |  |  |  |
| Hypoglycemia | 0 | 1(5.26%) | 0 |
| Total | 0 | 19 | 42 |
|  |  |  |  |
