# Supplementary material for: Efficacy of Berberine in Patients with Non-Alcoholic Fatty Liver Disease
Source: PLoS One. 2015 Aug 7;10(8):e0134172. doi: 10.1371/journal.pone.0134172 (PMC4529214; doi:10.1371/journal.pone.0134172)
Supplement: S1 Text — (DOCX) [file pone.0134172.s008.docx]

**Supplementary information**

**Methods**

**Real-time quantitative RT-PCR analysis**

Total RNA was isolated from liver tissues using Trizol reagent (Invitrogen, Carlsbad, CA). The cDNA was synthesized by reverse transcription using Rever-Tra Ace (Toyobo, Osaka, Japan). The SYBR Green PCR MasterMix (Toyobo, Osaka, Japan) was used for qPCR with a sequence detection system (ABI PRISM7900, Applied Biosystems, FosterCity, CA). The primers used for qPCR are shown as follows.

| Gene | Forward primer (5’- 3’) | Reverse primer (5’- 3’) |
| --- | --- | --- |
| MTTP | 5' CTC AAG GAG ATG ATT GTT CAC 3' | 5' CCA GAC CCA GAG TAG AGG ATA 3' |
| CPT1α | 5' CCA TCT CTT CTG CCT CTA TGT 3' | 5' GTC AGG GTT TTT CTC AAA GTC 3' |
| LDLR | 5' GCT GTG GGT TCC ATA GGG TTT C 3' | 5' TGC GGT CCA GGG TCA TCT TAC 3' |
| GcK | 5' CTT TTG AGA CCC GTT TCG TGT 3' | 5' GCC CCA GAG TGC TTA GGA TGT 3' |
| LPK | 5' CTT CAG CAC CAA CAG CAA ATC 3' | 5' GCC ATC AGC AGC AAT ACT CA 3' |
| Actin | 5' GAT TAC TGC CCT GGC TCC TA 3' | 5' TCA TCG TAC TCC TGC TTG CT 3' |

All samples were measured in triplicate with ratβ-actin as an internal control. Fluorescent signals were normalized to an internal reference (∆Rn), and the threshold cycle (Ct) was set within the exponential phase of PCR. The relative gene expression was calculated using the2 ^-∆∆ Ct^.

**Western immunoblot analysis**

Protein from rat liver samples was extracted with RIPA buffer [50 mmol/l Tris-HCl (pH 7.4), 1% NP-40, 0.5% sodium deoxycholate, 150 mmol/l NaCl, 0.1% SDS, EDTA, etc.] containing protease and phosphatase inhibitors. Protein concentrations were measured using a BCA-100 Protein Quantitative Analysis Kit. After denatured, protein samples were subjected to SDSPAGE and blotted onto polyvinylidene difl uoride (Millipore) membranes. Nonspecifi c binding sites were blocked with 5% skim milk in Tris-buffered saline containing 0.1% Tween 20 for

1 h and then incubated with primary antibodies against MTTP (No. BS6672,Bioworld), Glucokinase (No. 19666-1-AP, Proteintech), and CPT1α(No. 15184-1-AP) overnight at 4°C. After three washes in Tris-buffered saline containing 0.1% Tween 20, the membranes were incubated with horseradish peroxidaseconjugated secondary antibodies (anti-mouse or anti-rabbit IgG) for 1 h and visualized by ECL detection (Pierce Biotechnology, Rockford, IL). Quantitation was performed by Fujifi lm Las-3000 Luminescent Image Analyzer.

**LC–MS/MS Analysis**

A Shimadzu triple-quadruple MS (LC–MS/MS 8040; Shimadzu Corporation, Kyoto Japan) was applied for identification and quantification of BBR and its metabolites with a multiple reaction-monitoring mode (MRM). The tetrahydropalmaline served as an internal standard (m/z 356.0→192.0). Details in LC separation and ESI analysis were reported previously[19]. BBR working solution was made with methanol at concentrations of 0.05, 0.1, 0.05, 1.0 and 5.0 μg/mL. After weighing, liver samples were homogenized with 2 times volumes of saline. The blank rat liver homogenate (1 mL) was added with 100μL of BBR working solution to yield BBR concentration of 5.0, 10.0, 50.0, 100.0, 500.0 ng/mL, respectively; then the calibration curve were drawn based on values of these samples. Then, the study liver samples were prepared. 1.5 mL ethyl ether containing tetrahydropalmaline (50 μL, 1.40 μM) and sodiumhydroxide solution (50 μL, 0.5 M) was used to extract liver homogenate twice. The sample preparation thereafter was done as described previously[19]. An aliquot of 10 μL was injected into LC-MS/MS for analysis. By using calibration curve (Y = ax+ b), the concentrations of BBR and its metabolites (x (ng/g)) in the liver tissues were calculated as $x=\frac{V(y-b)}{a\cdot m}$ (y: Peak area ratios of the BBR or metabolite to internal standard; V (ml): volumes of residues reconstitution solution; m (g): the weights of each tissue).
